# Life in sediments fosters ‘sexual’ speciation in the *Shewanella baltica* complex

**DOI:** 10.1371/journal.pgen.1012306

**Published:** 2026-09-11

**Authors:** Víctor Fernández-Juárez, Francisco Salvà-Serra, Guillem Seguí, Alberto J. Martín-Rodríguez

**Affiliations:** 1 Department of Microbiology, Tumor and Cell Biology, Karolinska Institutet, Stockholm, Sweden; 2 Unit of Medical Technology and Biological Function, Department of Medical Technology and Diagnostics, RISE Research Institutes of Sweden, Mölndal, Sweden; 3 Department of Clinical Microbiology, Sahlgrenska University Hospital, Gothenburg, Sweden; 4 Culture Collection University of Gothenburg (CCUG), Sahlgrenska University Hospital and Sahlgrenska Academy, University of Gothenburg, Gothenburg, Sweden; 5 Department of Clinical Sciences, University of Las Palmas de Gran Canaria, Las Palmas de Gran Canaria, Spain; McGill University, CANADA

## Abstract

Understanding how intra- and interspecific differentiation arises in natural microbial populations is central to explaining the processes that drive bacterial evolution. Motivated by the co-occurrence of multiple putative genospecies closely related to *Shewanella baltica* in Baltic Sea sediments, we investigated the genomic structure of this species complex across fine spatial scales. We analyzed 112 genome sequences from strains collected across several sediment cores and depths (0–6 cm) at Vaxön (Stockholm archipelago, Sweden) as well as earlier isolates from this site and allopatric strains from surrounding locations obtained from both sediments and the water column. Using a reverse-ecology population genomics approach, we found unprecedented genomic diversification among sediment-associated strains, which form a species complex resolving into three cohesive evolutionary groups (G1, G2, and G3) with distinct signatures of metabolic specialization including sulfite respiration. While G1 consists predominantly of a single species (*S*. *baltica*) with high gene turnover, G2 and G3 comprise an array of divergent putative genospecies and previously reported species consistently recovered from sediments. Patterns of homologous recombination indicate that diversification of the lineages within G2 and G3 is primarily recombination-driven (‘sexual’) and is associated with specialization in sulfite reduction and utilization of certain carbon sources. The extent of diversity uncovered here far exceeds that reported for *S*. *baltica* from other environments, suggesting that a sediment-associated lifestyle promotes the emergence of novel genotypes. These findings expand the known limits of sympatric speciation in prokaryotes beyond subspecific ecotypes, demonstrating that bacterial species can diverge and persist as distinct lineages in the absence of spatial segregation and at microgeographic scales. Furthermore, our results suggest that collective interactions and ecological differentiation can structure sediment-associated bacterial populations strongly enough to drive divergence at the species level.

## Author summary

How new bacterial species arise remains one of the major questions in microbial evolution. Unlike plants and animals, bacteria exchange genes through homologous recombination, making it difficult to understand how genetically and ecologically distinct lineages can emerge while living together. We analyzed 112 genomes from *Shewanella* isolates collected from sediments at a site in the Baltic Sea, together with earlier local isolates and related strains from nearby sediment and water environments. We found that what was previously considered a single species, *S*. *baltica*, actually comprises three differentiated evolutionary groups occupying the same sediment habitat. Sediment-associated lineages appear to diversify through frequent homologous recombination while maintaining distinct evolutionary identities. These groups differ in their metabolic capabilities and patterns of gene exchange, suggesting that ecological specialization has contributed to their divergence. The remarkable diversity observed within a few centimeters of sediment greatly exceeds that reported for populations from other environments. Our findings show that bacterial populations can diversify into multiple species without geographic isolation, providing evidence that ecological differentiation and local interactions can promote speciation at extremely small spatial scales.

## Introduction

Defining how microbial diversity is structured is central to understanding microbial evolution. Analyses of bacterial whole-genome sequences and metagenome-assembled genomes have consistently shown that microbes tend to form sequence-discrete clusters with high intra-group average nucleotide identity (ANI), typically ≥95%, corresponding approximately to 70% of DNA-DNA hybridization (DDH) values [1], and separated by gaps in the range of 83–95% ANI [2]. While it remains an open question whether a continuum of genomic diversity among prokaryotes exists [3], these distinct clusters are broadly accepted as corresponding to what we refer to as ‘bacterial species’. Species circumscription thresholds are, however, somewhat blurry, with a frequently defined ‘gray zone’ around the 95–96% ANI window [4], and more stringent cutoffs (e.g., ANI ≥ 96% or taxon-specific thresholds) sometimes providing greater taxonomic coherence [5,6].

A challenge for the definition of bacterial species relies on how bacterial populations evolve. As asexual organisms, bacteria reproduce clonally by binary fission. Over time, DNA mutations may arise during DNA replication and lead to clonal speciation. Nevertheless, this appears to be a relative rarity in nature, as less than 10% of all bacterial species have been estimated to be truly clonal [7]. Instead, horizontal gene transfer (HGT) through various mechanisms has been consistently shown to represent, overall, a more powerful evolutionary driver [8–10]. Albeit HGT can occur across distant phylogroups, including across kingdoms [11,12], large-scale genetic exchange is rare between distant genomes [13]. Thus, analogously to the fundamentals (DNA recombination) and limits (interbreeding) of sexual reproduction in eukaryotes, barriers in large-scale HGT across bacteria could be regarded as species boundaries, and speciation driven by HGT is often referred to as ‘sexual speciation’ [14]. Indeed, the blurry ANI threshold delimitating species boundaries mentioned above has been proposed to emerge from the ANI limit (90–98%) in which gene flow is interrupted across distinct lineages [7]. Evidence suggests that HGT goes hand in hand with ecological adaptation, with HGT being promoted in bacterial populations sharing the same habitat or lifestyle [15–17]. This view is consistent with several speciation models that treat species as metapopulation lineages whose cohesion reflects both genetic exchange and shared ecological pressures, occupying different positions along a continuous speciation spectrum shaped by gene flow, recombination, and selection [18–21].

Renowned for their exceptional metabolic versatility, *Shewanella* species are ubiquitously distributed in water ecosystems and redox-stratified environments like aquatic sediments, where they play important roles in nutrient cycling and biogeochemistry [22–24]. The Baltic Sea, a semi-enclosed and highly stratified water body with sharp oxygen gradients across the water column and a longitudinal salinity gradient, is home to *Shewanella baltica*, a species that thrives under brackish conditions. Different *S. baltica* ecotypes, defined as independent monophyletic clusters occupying specific ecological niches [25], [26], have been described across the oxygen gradient of the water column [27]. These *S*. *baltica* populations undergo extensive homologous recombination, facilitating genetic exchange among strains, enhancing metabolic capabilities likely contributing to ecological fitness, and promoting strain diversification [28]. Although the high rates of HGT in *S. baltica* have been postulated to make ‘sexual’ speciation possible [27–31], factual evidence of new species formation has remained elusive.

Our team has devoted efforts to the genomic characterization of *Shewanella* spp. from water and sediments of the Baltic Sea and other aquatic environments around Stockholm, which have led to the identification of several novel species closely related to *S*. *baltica*, i.e., *S. scandinavica*, *S. vaxholmensis*, and *S. septentrionalis*, collectively referred to as the ‘*S*. *baltica* complex’, with *S. hafniensis* occupying a borderline position [32,33], Thus, intrigued by the co-occurrence of *S*. *baltica* and multiple intimately related novel genospecies in sediments, we set out to examine the genomic variation within this species complex. We examined the genomic content of arbitrarily selected strains from sympatric *Shewanella* populations sampled at different depths (0, 2, 4, and 6 cm) within the same sediment cores collected at Vaxön in the Stockholm archipelago. We also included strains obtained from earlier sampling at the same site and arbitrarily selected allopatric strains from other locations in the Stockholm region, resulting in a total dataset of 112 genome sequences. Using a comprehensive suite of state-of-the-art bioinformatic inferences, we delved into the genomic structure of the *S*. *baltica* complex and the evolutionary forces shaping and maintaining the observed phylogenetic and ecogenomic patterns. We demonstrate that bacterial speciation, that is, the emergence of stable strain lineages that are fully consistent with the standards for the designation of bacterial species [34], can be actively driven by recombination in sympatric populations at a microgeographical scale within a shared habitat. Here, we show an empirical model of ‘sexual’ speciation in natural bacterial communities shaped by a sediment-associated lifestyle is a key driver of this process.

## Results

### Surficial sediments in Vaxön are taxonomically and functionally homogenous

Our study began with the collection of four surficial sediment cores, termed SP0, SP1, SP2, and SP4 (~7–8 cm deep) from the shoreline of Vaxön, an island in the Stockholm archipelago (12 August 2023; S1 Fig). Environmental DNA was extracted from sediments at 0, 2, 4, and 6 cm depths for metagenomic analysis. Across these depths, metagenomics revealed no significant changes in bacterial community composition or functional pathways (S2A- S2E Fig; *p* > 0.05). *Actinobacteriota* dominated the communities (18.1-38.5% of reads), followed by *Pseudomonadota* (17.0-25.0%), *Bacteroidota* (1.3-2.4%), and *Planctomycetota* (1.0-2.2%) (S2A Fig). Neither alpha- nor beta-diversity, assessed using Shannon indices and Euclidean distances, respectively, differed significantly with depth (*p* > 0.05; S2A, S2B Fig). Functional annotation of metagenomic reads based on KEGG pathways showed no depth-dependent clustering (*p* > 0.05, Kruskal-Wallis test; S2C, S2D Fig). Predominant pathways (log_10_ GPM > 7) included ABC transporters, quorum sensing, DNA replication and repair, glycolysis/gluconeogenesis, fatty acid metabolism, and purine metabolism (S2E Fig). *Shewanella* spp. populations accounted for 0.04-0.21% of total bacterial reads (S1 Table), representing more than 2.3% of *Gammaproteobacteria* and ranking among the 100–300 most abundant genera detected (out of >13,000). However, *Shewanella* abundance did not differ significantly across sediment depths (S2F Fig; *p* > 0.05). Together, these data indicate that (i) bacterial community composition remains largely unaltered across the upper 6 cm of sediments, and (ii) *Shewanella* is not a particularly dominant taxon in the surficial sediment community.

### A sympatric lifestyle within surficial sediments fosters genotypic divergence in *Shewanella* populations

From four sediment cores collected at the shoreline in Vaxön, we isolated 60 randomly selected coexisting *Shewanella* strains, i.e., sympatric (designated with the prefix ‘VAX’; S2 Table). We complemented this collection with 18 *Shewanella* strains obtained during previous isolation efforts in the same area in 2021 (prefix ‘SP’), as well as randomly selected allopatric strains from sediment (n = 19) and water (n = 15) samples collected from distinct locations in and around Stockholm between 2021 and 2023. These sites included Nynäshamn (n = 11; prefix ‘N’), Tyresö (n = 12; prefix ‘T’), Lidingö (n = 8; prefix ‘L’), and Hagaparken (n = 3; prefix ‘H’) (S1 Fig). In total, we analyzed 112 *Shewanella* whole-genome sequences, including previously sequenced strains [32,33]. Of these, 32 were complete genomes, all belonging to representative members of the *S. baltica* complex (S2 Table).

**Fig 1:**
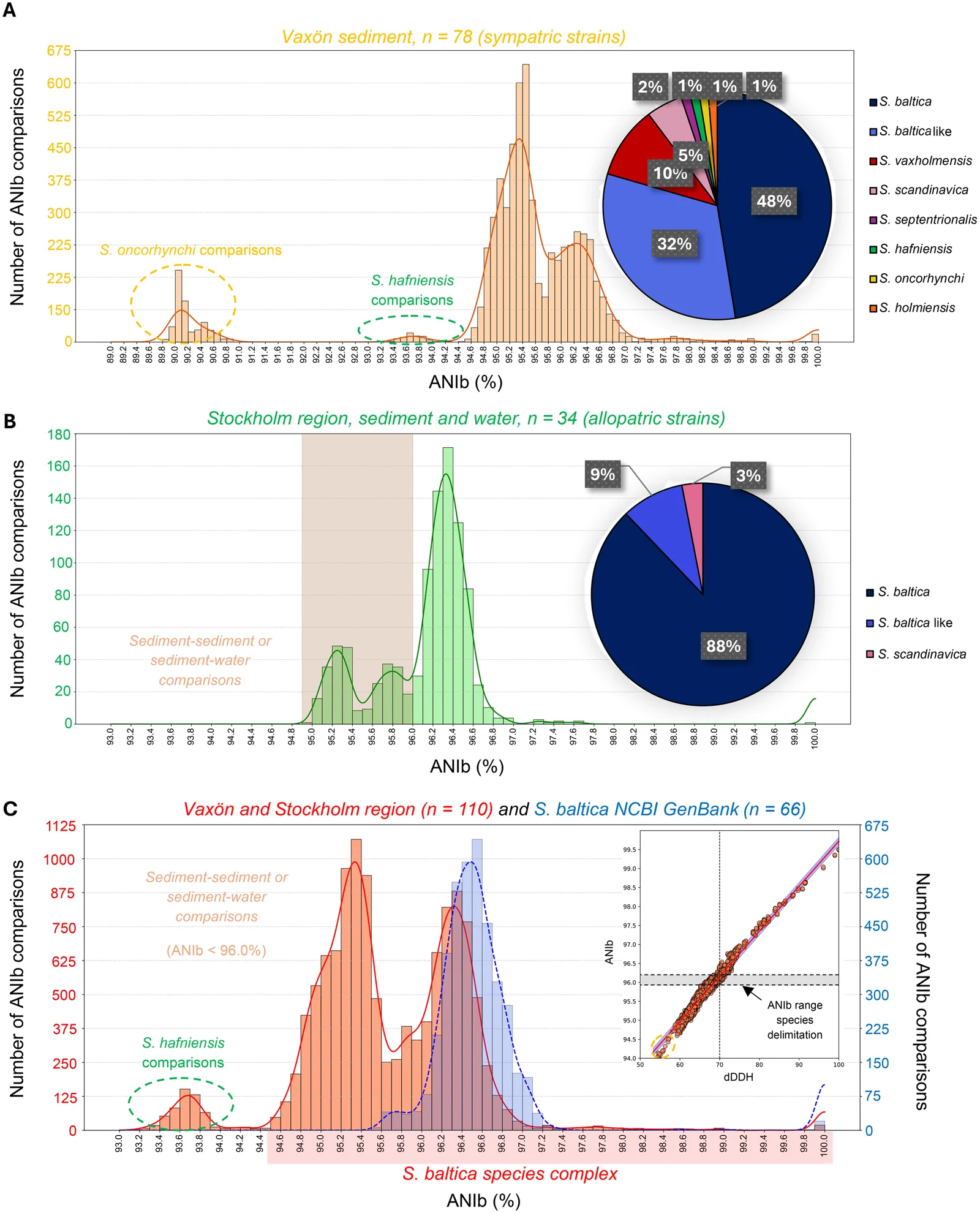
Genomic diversity of *S. baltica* complex genomes based on ANIb relatedness. **(A-C)** Frequency distributions of pairwise ANIb comparisons (0.1% windows) for (**A**) strains recovered from Vaxön sediment cores in 2023 (n = 60) and from previous sampling occasions in the same region (n = 18); (**B**) allopatric strains retrieved from other regions in or around Stockholm city: sediments (n = 19) and water (n = 15); and **(C)** the entire dataset, including both our isolates (108 *S. baltica* complex members and 1 *S. hafniensis*, excluding *S. oncorhychi*) along the type strain *S. baltica* CECT 323^T^ (in red), and those available from NCBI GenBank (n = 66, shown individually in blue for comparison). In **(A)**, dashed areas denote comparisons with the species most closely related to the *S. baltica* complex strains recovered in our study, i.e., *S. oncorhynchi* (n = 3) and *S. hafniensis* (n = 1)*.* In **(A-B)**, pie charts showing the affiliation of the isolates as determined by TYGS were added as insets. In (**B),** the brown frame highlights the ANIb comparisons between strains recovered from the sediment. In **(C)**, the all-vs-all genome-to-genome distance calculator (GGDC) analysis is embedded within the ANIb comparison, showing that ANIb values corresponding to the 70% digital DNA–DNA hybridization (dDDH) species delineation threshold fall within 95.8–96.1%, thereby defining species boundaries within the species complex.

To assess the preliminary taxonomic diversity of the *Shewanella* isolates retrieved from Vaxön, comprising 78 isolates from the 2021 and 2023 sampling occasions combined, whole-genome sequences were submitted to the Type Strain Genome Server (TYGS) [35], which uses 70% digital DNA-DNA hybridization (dDDH) as the threshold for species designation. Based on this indicator, 47% of the isolates were affiliated with *S. baltica* (n = 37), 10% to *S*. *vaxholmensis* (n = 8), 5% to *S*. *scandinavica* (n = 4), and 1% to *S. septentrionalis* (n = 1)*,* whereas a remarkable 32% could not be confidently assigned to any known species (n = 25), but exhibited the closest similarity to *S. baltica* (Fig 1A). Thus, ~ 95% of the Vaxön isolates in this dataset were taxonomically very closely related, with the remaining strains affiliated with *S. hafniensis*, *S. oncorhynchi*, and *S. holmiensis* (Fig 1A). Pairwise ANIb comparisons between all these strains exhibited values that ranged from 89.6% to 100%, with two clear ANIb gaps observed between the interactions *S. oncorhynchi* vs. *S. hafniensis* (91.0-93.2%) and *S. hafniensis* vs. *S. baltica* complex members (94-94.5%) (Fig 1A)*.* Of note, we found a distinct pattern within the *S*. *baltica* complex characterized by a continuous bimodal distribution with 60% (3,743 pairwise comparisons) falling within the 94.5%-95.9% range, while 28% (1,748 comparisons) clustered between 95.9%-97.2% (Fig 1A).

We extended our analysis to a regional set of 34 *S. baltica* complex strains isolated from sediment and water samples collected at distinct locations across Stockholm. Given their geographic separation from the strains presented above, we treated them as allopatric strains (S1 Fig). In this set, 88% of the strains (n = 29) could be unambiguously affiliated to *S. baltica* by TYGS*,* whereas 9% (n = 3) could not be affiliated to a known species, and 3% (n = 1) circumscribed within *S*. *scandinavica* (Fig 1B). Here, the ANIb distribution revealed a trimodal pattern with 15.4% of interactions (168 pairwise comparisons) falling within the 94.8%-95.5% range, 12.4% (135 pairwise comparisons) within 95.5%-96.0%, and 68.2% (743 pairwise comparisons) within 96.0%-97.0% (Fig 1B). The first two ANIb peaks reflected interactions between sediment-sediment or sediment-water genome pairs, whereas the highest density peak (96.0%-97.0%) was observed exclusively among water-water genome comparisons, suggesting a potential role of sediments in driving genomic diversification.

ANIb pairwise comparisons across the integrated Vaxön (sympatric) and Stockholm (allopatric) genome sequence dataset corroborate the observed bimodal pattern, with high average shared genome fractions (84.8 ± 3.63%; S3A Fig). The lower ANIb peak (94.5-95.6%) accounted for ~50% of comparisons (n = 6,383), mostly involving sediment-derived isolates, with the sole exception of *S. septentrionalis*, isolated from surficial shore water near the sediment layer [33]. Notably, no *Shewanella* genome sequences from NCBI GenBank fell within this range (reviewed on 31 May 2025, S3 Table), highlighting the novelty of this population. The higher ANIb peak (centered ~96.3%) encompassed 32% of comparisons (n = 4,148), predominantly *S. baltica* strains, closely overlapping publicly available *S*. *baltica* genomes (~96.5% ANIb; Fig 1C). This range, bordering the canonical ~96% species delineation threshold, was benchmarked against FastANI-based intra-specific comparisons within other well-represented *Shewanella* clades (*S. algae*, 219 genomes; *S. xiamenensis*, 98 genomes; *S. oncorhynchi*, 32 genomes; S3 Table), which displayed unimodal FastANI distributions with narrower dispersion and consistently higher intra-specific similarity (>97–98% FastANI). In contrast, *S*. *baltica* (including both our isolates and publicly available genome sequences) spanned a broader FastANI range, extending toward the species boundary zone, and reflecting the exceptional intra-specific diversity of *S. baltica* (S3B Fig). To define species boundaries within the *S. baltica* complex, we determined the ANIb values corresponding to the 70% dDDH species threshold, which fell within 95.8-96.2% (Fig 1C, inset). Of note, more than half of all pairwise ANIb values fell within 94.5-96.2%, a transition zone consistent with the typical ANI gap for species delineation [4]. Taken together, these patterns represent a possible case of ongoing sympatric speciation in sediments.

### Multiple differentiated lineages coexisting in surficial sediments provide a snapshot on *Shewanella* speciation

**Fig 2:**
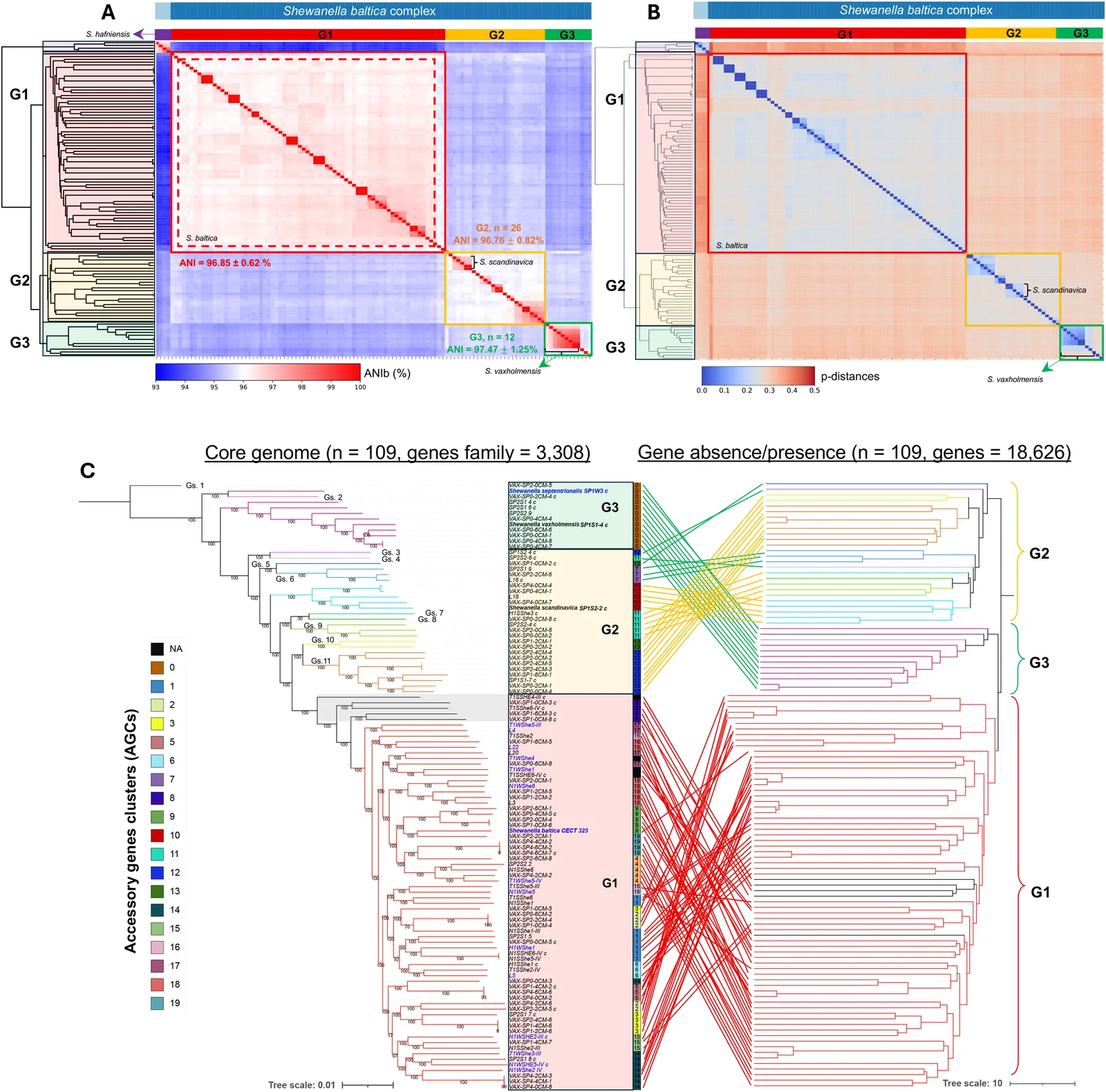
ANI clustering, genome-wide, and pangenome analysis of the *S*. *baltica* complex from the Stockholm region. **(A)** Heatmap of all-vs-all ANIb values for the *S. baltica* complex. The dashed red frame within G1 denotes the strains affiliated with *S. baltica*. **(B)** Heatmap showing pairwise genome-wide differentiation between strains, based on SNPs extracted from whole-genome sequence alignments. SNPs were grouped into overlapping 10 kb sliding windows with a 2.5 kb step size. For each window, the proportion of SNPs with discordant genotypes (*p*-distance) was calculated for all genome pairs, producing normalized values from 0 (identical) to 0.5 according to the genomic divergence. In (**A**) and **(B)**, as the closest relative, *S. hafniensis* (strain SP1S1-9 and three additional sequences from NCBI GenBank, S3 Table) was added as an outgroup, represented in purple. For clarity, the plots indicate the previously characterized species within the complex (*S. baltica*, *S. scandinavica*, and *S. vaxholmensis*). **(C)** Combined core genome and accessory genome tree constructed using a 90% identity and 90% coverage threshold based 3,310 core gene and protein families from the 109 genomes (including *S. baltica* CECT 323^T^ as reference). For building the accessory tree, pairwise Euclidean distances between isolates were calculated, and an average-linkage (UPGMA) method was applied. Blue labels indicate genomes isolated from the water column. Within G2 and G3, branch colors indicate distinct species previously described, as well as the genospecies (“Gs.”). Species and genospecies were delineated as explained in the main text. Accessory gene groups (AGCs) are indicated by distinct numbers and colors. Lines link corresponding genomes between the core genome and accessory genome trees, illustrating their topological relationships. Numbers on the branches denote bootstrap support values after 1,000 replications. In all the plots, groups are differentiated by color, i.e., red for G1, yellow for G2, and green for G3. The grey shadow within G1 denotes incipiently divergent genomes that could not be affiliated to *S. baltica* or differentiate as putative genospecies.

To assess the extent of phylogenomic differentiation within our dataset, we performed pairwise ANIb and dDDH comparisons and reconstructed phylogenies based on the core genome and core proteome content. Genomic species boundaries were assessed based on current standards [34], using the following criteria: (i) ANIb ≥ 95.8-96.2% (all vs. all); (ii) dDDH ≥ 70% (all vs. all); and (iii) phylogenomic coherence between core genome and core proteome trees. All-against-all ANIb comparisons defined three main groups (G), namely, G1, comprising mostly *S. baltica* strains plus several unassigned strains; G2, closely related to *S. scandinavica*; and G3, related to *S. vaxholmensis* and *S. septentrionalis* (Fig 2A). ANIb values between groups showed clear boundaries supporting this clustering (<96%), with intergroup similarities ranging from 94.89-95.97% (G1 vs. G2), 94.50-95.91% (G1 vs. G3), and 95.10-95.71% (G2 vs. G3) (Fig 2A). Genome-wide differentiation within the *S. baltica* complex was assessed using SNP-based sliding window analyses, and pairwise *p*-distances ranged from 0 to 0.5 (Fig 2B). Values above 0.25 indicate high genomic differentiation between the defined groups, whereas values near 0 within G2 and G3 indicate close genomic relatedness and support the emergence of new genomic species clusters within these groups (Fig 2B), aligning with the ANIb group classification.

Our phylogenomic reconstructions, based on both core genome and core proteome content, comprising 3,308 core family genes (3,399,949 bp) or proteins (1,131,112 aa) from a pangenome of 18,626 genes (representing ~75% of the core genes in each genome), were highly congruent with one another and their topology was consistent with inferences drawn from overall genome relatedness indexes (OGRIs) previously introduced (Figs 2C, S4A). In total, we identified 11 putative novel genospecies (referred to as ‘genospecies’ hereafter) within G2 and G3, specifically, 9 within G2 and 2 within G3 (Fig 2C). Each lineage was genomically differentiated both from one another and from the previously proposed species *S. septentrionalis*, *S. vaxholmensis*, and *S. scandinavica* [32,33]. Several strains from different time points (2021 - ‘SP’ prefix, and 2023 - ‘VAX’ prefix) were affiliated with the same species or genospecies, e.g., various *S. vaxholmensis* strains (G3) and diverse isolates belonging to a genospecies within G2, e.g., genospecies 11 (Fig 2C). This finding evidenced stability over time of divergent lineages in this sediment ecosystem. In contrast, strains showed no clear grouping by geography or sediment depth (Fig 2C), consistent with their widespread distribution, and the taxonomic and functional homogeneity inferred from our metagenomic analyses. Thus, our phylogenomic analyses show that sympatric diversification within the *S. baltica* complex might be driven by the close coexistence of strains in sediments, which could be promoted by extensive lateral gene exchange in this habitat.

### High rates of lineage-specific gene turnover and HGT drive accessory genome diversification in the *S. baltica* complex

**Fig 3:**
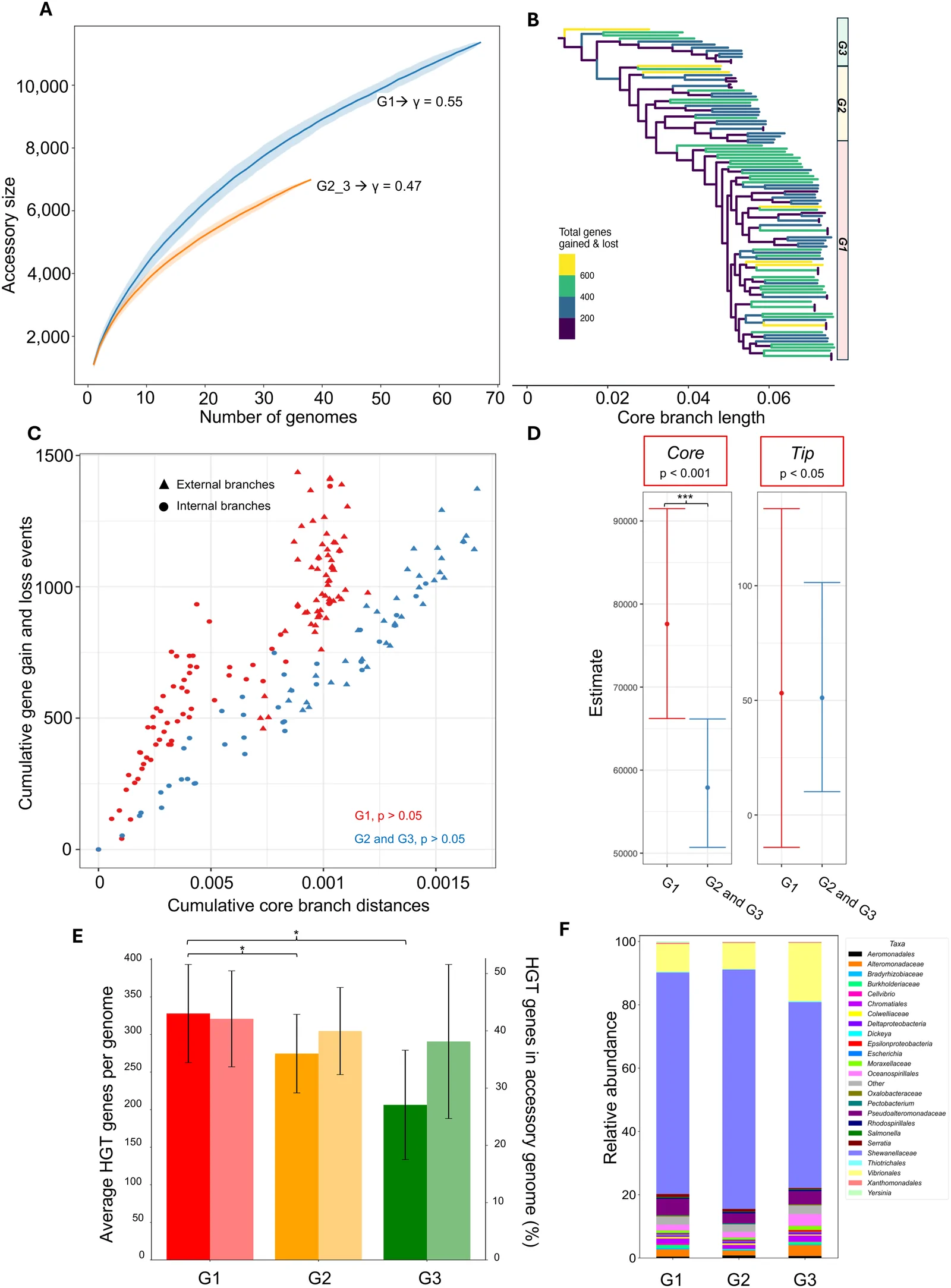
Gene gain, loss, and HGT patterns in the *S. baltica* complex pangenome. **(A)** Accumulation curves of accessory genome content of the members of the *S. baltica* complex from this study (n = 109). Heap’s Law exponent (γ) was fitted to the cumulative accessory genome for each dataset. **(B)** Core genome tree, with branches colored according to the number of gene gain and loss events inferred using maximum parsimony. **(C)** Cumulative number of gene gain/loss events plotted against cumulative branch length, and (**D**) gene exchange parameters estimated by Panstripe. ‘Core’ reflects the association between gene gain/loss events and core phylogeny branch length, and ‘tip’ indicates gene exchange events occurring specifically at terminal branches. The *p* value within the ‘core’ and ‘tip’ boxes indicates the statistical significance of turnover events associated with the core and the tip in all the groups compared. In **(A)**, **(C)**, and **(D)**, to minimize potential biases in comparisons, we merged G2 and G3 (n = 38), which, although evolutionary distinct, displayed significantly lower accessory content and rates of gene turnover compared to G1. **(E)** Number of HGT genes per genome (left bar) and the percentage of HGT genes relative to the total accessory genome of each genome (right bar) across the indicated groups. **(F)** Taxonomic classification of genes detected within RGPs, assigned using EggNOG, across the different groups. For **(D)** and **(E)**, statistically significant differences are indicated by asterisks: *p* < 0.05 (*) and *p* < 0.001 (**).

To contextualize gene exchange in the *S. baltica* complex, we assessed the distribution of accessory genes across the phylogeny. We integrated an accessory genome tree derived from gene presence/absence profiles with the core genome phylogeny, which revealed distinct and group-specific accessory gene clusters (AGCs) which, overall, were congruent with the topology of the phylogenomic reconstructions (Fig 2C). Strains within G3 shared a highly similar AGC, whereas nearly every species and genospecies cluster within G2 exhibited a distinct AGC from one another. In contrast, G1 strains displayed a much higher diversity of AGCs, up to 16, which did not consistently align with the core genome tree topology (Fig 2C). The high number of AGCs observed in the *S. baltica* complex is consistent with its evidently open pangenome, encompassing up to 87% of the total pangenome and including over 15,000 accessory genes (Fig 3A), constituting ~25% of each genome. Despite G1 forming a cohesive cluster primarily composed of a single species, *S. baltica*, it harbored substantially greater accessory genome diversity than G2 and G3 combined, with Heap’s Law exponent (γ) fitted to the cumulative accessory genome curve of 0.55 for G1, compared to 0.47 for G2 and G3, confirming a more open pan genome in G1 (Fig 3A). Furthermore, the accessory genome of the *S. baltica* complex exceeded that of other *Shewanella* species such as *S. algae* and *S. xiamenensis*, and was comparable to that of *S. oncorhynchi* (S4B Fig).

We next studied gene turnover in *S. baltica* complex using the Panstripe pipeline [36]. Overall, the *S. baltica* complex exhibits 200-800 gene gain/loss events per genome (Fig 3B), with G1 showing significantly more events (352 ± 124) than G2 and G3 (250 ± 168; *p* < 0.05; Fig 3B), consistent with its broader accessory gene content (Figs 2C, 3A). Older (internal) and newer (external) lineages displayed similar rates of gene turnover along their corresponding phylogenetic branches (*p* > 0.05; Fig 3C), yet a pronounced ‘tip effect’ was detected across the complex, with turnover events concentrated on terminal branches, where younger lineages exchange genes more actively than older ones (*p* < 0.05; Fig 3D). This elevated turnover is also linked to core genome divergence (*p* < 0.001; Fig 3D), where G1 shows significantly higher core genome divergence from G2/G3 (*p* < 0.05; Fig 3D), reinforcing that genetically distinct lineages drive the greatest gene flux. To better understand this, we explored the regions of genome plasticity (RGPs), and observed that approximately 40% of accessory genes were located within putative RGPs, with each genome containing 140–380 putative HGT-derived genes (Fig 3E). Across all groups, the majority of RGPs were attributed to the family *Shewanellaceae* (58.7–75.6%), followed by diverse aquatic bacterial taxa including *Vibrionales*, *Pseudoalteromonadaceae*, *Alteromonadaceae*, and *Oceanospirillales* (S5B Fig). G1 contained significantly more HGT-derived genes (328 ± 65 per genome) than G2 and G3 (250 ± 65 per genome; *p* < 0.05; Fig 3F), consistent with its more open pangenome.

Plasmids can drive ecological adaptations and even speciation in bacteria [37]. Here, we recovered 21 complete circular plasmids (2,677–120,678 bp) from 32 complete *S. baltica* complex genomes, and 15 genomes carried 1–4 plasmids (S4 Table). Closely related plasmids isolated in 2021 and 2023 suggest plasmid stability in the sediment ecosystem (S5A Fig). A few plasmids clustered together based on FastANI relatedness, but these clusters spanned all strain groups (S5A Fig), indicating limited group-specificity. Plasmids were enriched in replication, mobilome, transcription, vesicular transport, and defense functions (S5B Fig), though most plasmid genes remain uncharacterized (S4 Table). Given their limited group specificity and functional profile, plasmid-borne genes are unlikely to be major drivers of accessory genome differentiation in the *S. baltica* complex. This indicates instead that horizontally acquired chromosomal genes represent the main source of genomic functional diversification, likely contributing to the ecological specialization of distinct lineages within the described groups through the acquisition of niche-specific traits.

### Metabolic and ecological specialization differentiates groups within the *S. baltica* complex

**Fig 4:**
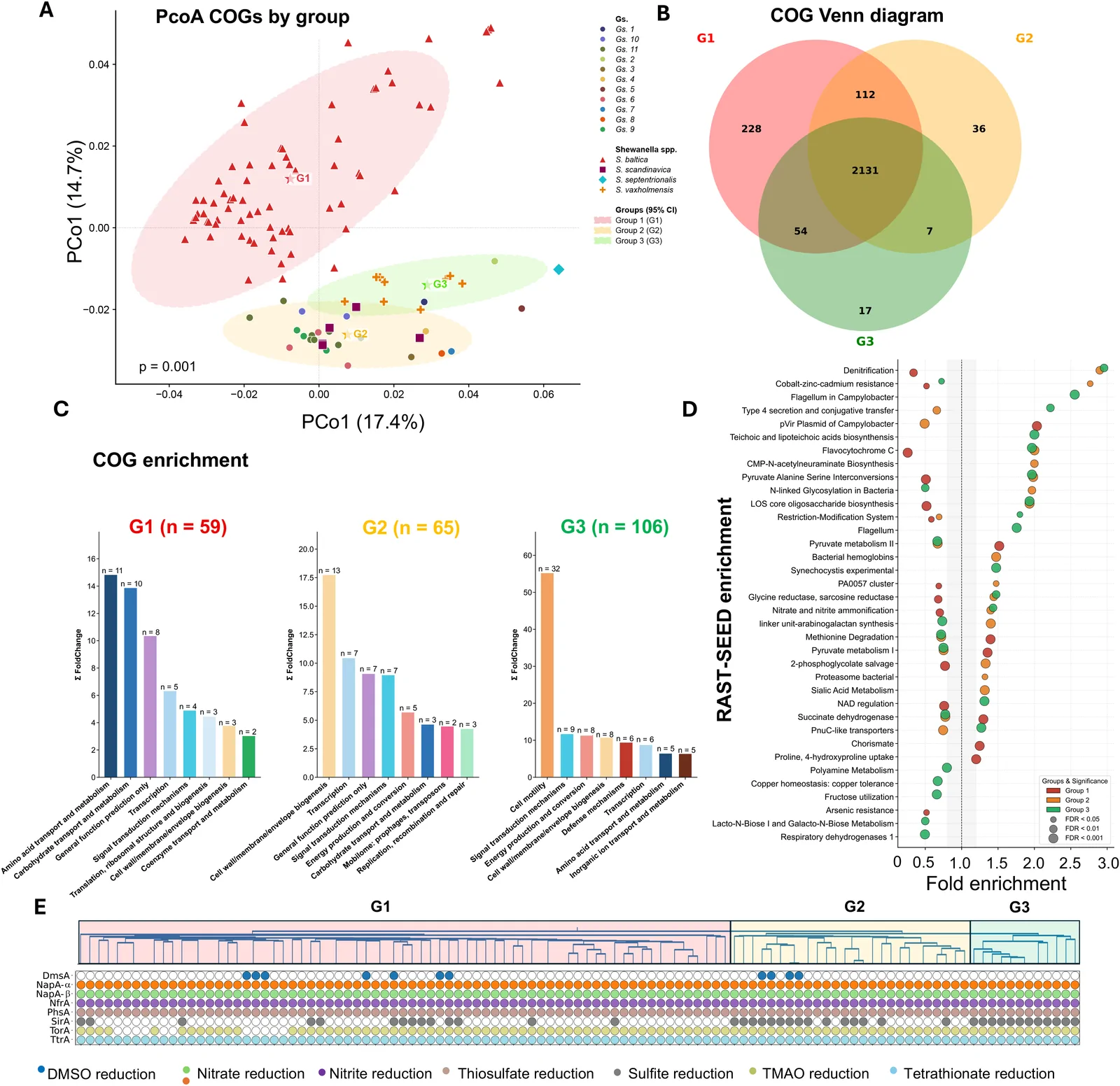
Metabolic specialization across the *S. baltica* complex. **(A)** Principal Coordinate Analysis (PCoA) of COG functional profiles for all strains, colored by phylogenetic group (G1, red; G2, yellow; G3, green), with 95% confidence ellipses shown. Species and genospecies-level groupings are indicated by point shapes. Groups are significantly differentiated (PERMANOVA, *p* = 0.001). **(B)** Venn diagram depicting the distribution of COGs across the three groups. The exclusive COGs by group are listed in S5 Table. **(C)** COG category enrichment analysis showing significantly enriched functional categories (FDR-adjusted *p* < 0.05) for each group, with the total number of enriched COGs indicated above each panel. **(D)** RAST-SEED pathway-level enrichment analysis showing fold enrichment of significantly enriched metabolic pathways across groups (FDR-adjusted *p* < 0.05). The specific enriched COGs and SEED annotations by group are listed in S6 and S7 Tables. **(E)** Distribution of genes encoding reductases for alternative electron acceptors (AEAs) across all sequenced genomes, organized by phylogenetic groups. Presence/absence patterns are shown for DMSO (DmsA), nitrate (NapA orthologs α and β), nitrite (NrfA), thiosulfate (PhsA), sulfite (SirA), TMAO (TorA), and tetrathionate (TtrA) reductases. Proteins were grouped and color-coded according to which group of reductases they were affiliated by these functional categories as detailed in the legend. Genomes along the x-axis are ordered according to their ANIb relatedness.

We investigated genomic signatures of ecological specialization by characterizing the functional genomic repertoire of each phylogenetic group through a cluster of orthologous groups (COG) analysis. PCA of functional profiles revealed three distinct, well-separated clusters, indicating functional differentiation between groups (PERMANOVA, p = 0.001, Fig 4A). All groups shared a large core of 2,131 COGs, yet several exclusive COGs were found in each group (Fig 4B, S5 Table). G1 had the largest exclusive set with 228 COGs, consistent with the high dispersion observed in the PCA plot for this group, its large accessory genome, and its elevated gene turnover, followed by G2 with 36 COGs and G3 with 17 COGs (Fig 4B, S5 Table). Pairwise comparisons further showed 112 COGs shared exclusively between G1 and G2, 54 between G1 and G3, and only 7 between G2 and G3 (Fig 4B). Within G2, no significant functional differences were observed among constituent species or genospecies (PERMANOVA, *p* > 0.05, Fig 4A), whereas G3 exhibited significant different functional profile between its constituent species and genospecies (PERMANOVA, p = 0.001; Fig 4A).

While KEGG pathway analysis revealed no overall metabolic differences among groups, except for galactose biosynthesis and degradation pathways, which showed greater completeness in G1 (S6 Fig), group-specific functional enrichment at both the COG category and pathway levels (using the RAST-SEED annotation) revealed distinct metabolic signatures across the three groups (FDR-adjusted *p* < 0.05; Fig 4C, 4D; S6, S7 Tables). G1 was enriched in 59 COGs predominantly linked to amino acid transport and metabolism (n = 11) and carbohydrate transport and metabolism (n = 10), which at the pathway level were associated with significant enrichment in pyruvate metabolism, methionine degradation, and succinate dehydrogenase activity (Fig 4C, 4D; S6, S7 Tables). In contrast, G2 and G3 shared enrichment in nitrogen metabolism, pyruvate-alanine interconversions, and biosynthetic pathways for cell surface structures such as lipo-oligosaccharide core oligosaccharides (Fig 4D; S6, S7 Tables). Beyond this shared profile, each group showed distinct specializations, and G2 was enriched in 65 COGs associated with cell wall/membrane biogenesis (n = 13), transcription (n = 7), and signal transduction (n = 7), complemented by pathway-level enrichment in heavy metal resistance, CMP-*N*-acetylneuraminate biosynthesis, or arabinogalactan synthesis (Fig 4C,4D; S6, S7 Tables). G3 displayed the broadest enrichment with 106 COGs, dominated by cell motility (n = 32), signal transduction (n = 9), energy production (n = 8), and defense mechanisms (n = 6) with corresponding pathway-level enrichment in flagellar biosynthesis, type IV secretion systems, and restriction-modification systems (Fig 4C, 4D; S6, S7 Tables). Together, despite the isolation of strains from all three groups across all sediment depths and the lack of significant variation in community composition or bulk biological functions across the upper 6 cm of sediment, each group within the *S*. *baltica* complex exhibits distinct genomic signatures consistent with metabolic specialization, suggesting distinct ecological strategies within a shared habitat.

**Fig 5:**
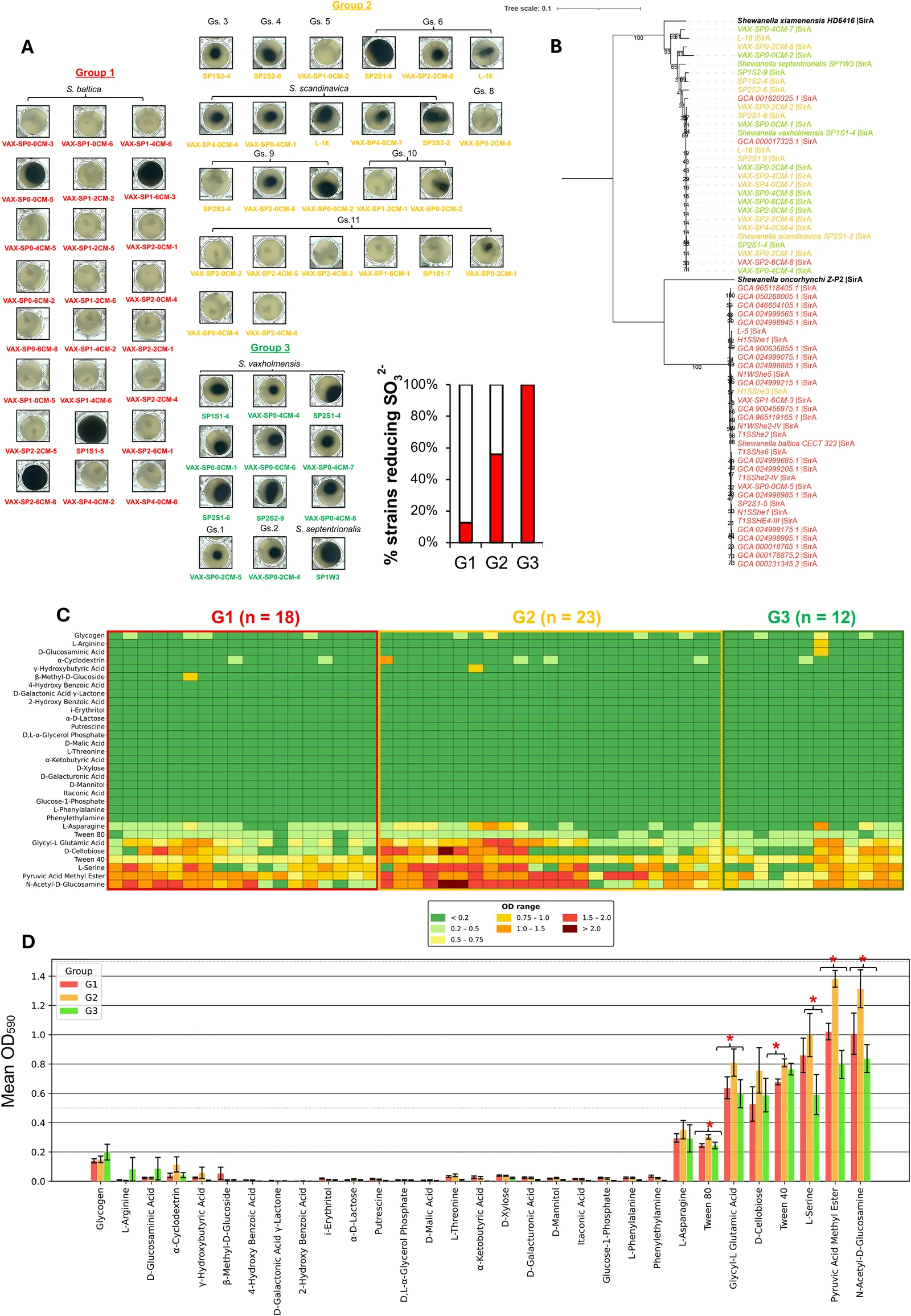
Ecological specialization across the groups of the *S. baltica* complex. (**A)** Phenotypic assessment of sulfite reduction capacity across strains from G1 (n = 40), G2 (n = 25), and G3 (n = 12), organized by species and genospecies. Images from H_2_S detection agar medium show growth on sulfite-containing media, where a dark precipitate indicates a positive sulfite reduction phenotype. Twenty-four representative G1 strains, and all G2 and G3 strains are shown. The stack-bar chart summarizes the proportion of sulfite-reducing (positive, red) and non-reducing (negative, white) strains per group. **(B)** Phylogenetic tree of SirA sequences retrieved from *S. baltica* complex genomes (S9 Table). **(C)** Heatmap of carbon source utilization profiles across representative strains from G1, G2, and G3, assessed using Biolog EcoPlates. **(D)** Mean optical density (OD_590_) per carbon source across groups. Asterisks indicate significant differences between groups, as determined by pairwise Wilcoxon tests after a Kruskal-Wallis test (*p* < 0.05).

To explore these strategies, we next focused specifically on energy metabolism, examining genes encoding reductases for alternative electron acceptors (AEAs) to evaluate potential differences in respiratory capacities. BLASTp searches were performed using reference *S. baltica* complex reductase catalytic subunits for nitrate (NapA isoforms α and β), nitrite (NrfA), thiosulfate (PhsA), sulfite (SirA), tetrathionate (TtrA), dimethyl sulfoxide (DMSO; DmsA), and trimethylamine *N*-oxide (TMAO; TorA) against all sequenced genomes (Fig 4E, see Materials and Methods). Genes for nitrate, nitrite, tetrathionate, and thiosulfate reduction were conserved across all genomes (Fig 4E). We observed loss of TMAO reductase genes in distinct G1 strains, as well as acquisition of DMSO reduction genes in multiple G1 and G2 strains (Fig 4E; S8 Table). The *sirA* gene, encoding a sulfite reductase, was conserved in G3 (100%) but was only present in 56% of G2 members and 21% of G1 strains. Extension of this analysis to public G1 genome sequences revealed a similar proportion (26.5% of genomes carrying *sirA*), which indicates this is a globally extended characteristic within the G1 lineage. Synteny analysis showed that *sirA*, which is part of a large operon (*sirABIGCDJKLM*), is located in a RGP together with the transcriptional regulator *sirR* and the inversely transcribed *sirEFG* operon encoding a specific heme lyase required for SirA maturation [38] (S7 Fig), which altogether suggests a functional sulfite reduction gene cluster hereafter referred to as the *sir* cassette. We experimentally validated this genomic prediction, assessing the sulfite reduction capacity by growing representative strains in H_2_S detection agar medium. The phenotypic results confirmed genome sequence-derived inferences across groups (Fig 5A).

Notably, phylogenetic analysis of SirA protein sequences (including G1 strains from the NCBI GenBank dataset) revealed two distinct clades (Fig 5B). One comprised the G2 and G3 SirA proteins, whose closest ortholog was the *S. xiamenensis* SirA protein (98.31-98.45% sequence identity), whereas G1 SirA proteins formed a separate clade with that of *S. oncorhynchi* as their closest ortholog (92.87-93.00% sequence identity, Fig 5B). Only two G1 strains from the NCBI GenBank dataset (genome accessions GCA_001620325.1 and GCA_000017325.1) had the *S. oncorhynchi* ortholog as their closest SirA relative, both originating from the Baltic Sea. The accessions for building the phylogenetic tree are disclosed in S9 Table. At the DNA sequence level, the *sir* cassette in G2 and G3 strains shared 83% sequence identity with the corresponding cassette in *S*. *xiamenensis*, whereas the G1 cassette shared 79% identity with that of *S*. *oncorhynchi*. The *S*. *oncorhynchi* cassette lacks two genes present in the G1, G2, and G3 cassettes, arguing against derivation of the G1 cassette from *S*. *oncorhynchi* (S7 Fig). The genomic regions flanking the *sir* cassette showed conserved synteny across G1, G2, and G3 strains, as well as *S*. *xiamenensis* and *S*. *oncorhynchi*, which is compatible with recombination in shaping the distribution of these cassettes within the *S*. *baltica* complex. Known transposases were rarely detected in the vicinity of *sir* cassettes, providing limited support for transposase-mediated mobilization (S7 Fig).

We further expanded our analysis linking genomic differentiation with metabolic specialization by assessing carbon source utilization across representative strains using Biolog EcoPlates (G1, n = 18; G2, n = 23; and G3, n = 12). While most substrates were utilized similarly across groups, G2 strains consistently showed higher utilization of *N*-acetyl-D-glucosamine (consistent with their enrichment in CMP-*N*-acetylneuraminate biosynthesis COGs, Fig 4D) as well as pyruvic acid methyl ester, L-serine, glycyl-L-glutamic acid, Tween 40 and 80, D-cellobiose, and L-asparagine (Fig 5C, 5D). These results suggest that metabolic diversification extends beyond sulfite reduction to carbon metabolism.

### Recombination drives divergence within the *S. baltica* complex

**Fig 6:**
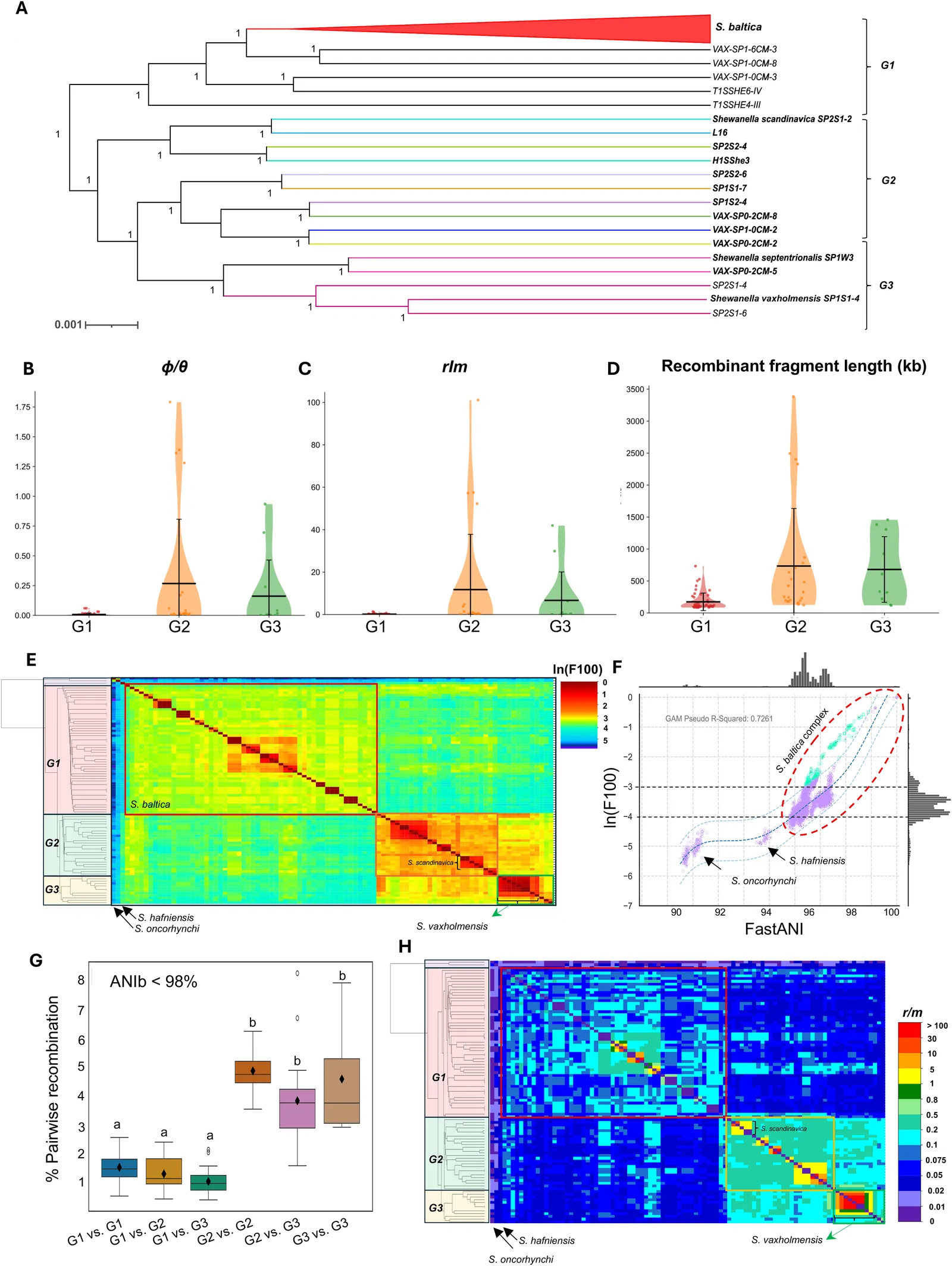
Recombination events within sediment strains of the *S*. *baltica* complex from the Stockholm archipelago. **(A)** Unrooted Bayesian phylogeny tree based on core-proteome sequences of representative closed genomes of the *S. baltica* complex (n = 32). *S. baltica* subspecies were collapsed.Node labels indicate highest posterior density values, and bold labels denote the different genospecies. **(B)** Gubbins-based recombination statistics shown as violin plots for each phylogenetic group, displaying the ratio of recombination to mutation rate (ϕ/θ), (**C**) the ratio of recombination events to mutation events (*r/m*), and (**D**) recombinant fragment length (bp). **(E)** F100 clustered heatmap showing the frequency of genes that are 100% identical between genome pairs, as a proxy of potential recombining genes. **(F)** F100 expected for their ANI range, i.e., F100 vs FastANI, building a GAM model using the sediment genomes (r^2^ = 0.72). The dashed red circle denotes the members of the *S. baltica* complex, and turquoise dots highlight genomes that do not follow the GAM model. **(G)** Percentage of recombination between pairwise genomes among the different groups within the *S. baltica* complex with ANIb < 98%. Different letters indicate statistically significant differences between groups (*p* < 0.05), while groups sharing the same letter do not differ significantly (*p* > 0.05). **(H)** Pairwise estimation of *r/m*. In (**E**) and **(F)**, F100 values were ln-transformed (ln[F100]) to improve visualization across the full range of values. In (**E**) and (**H**) different colors and frames represent the groups as previously described, indicating the previously characterized species within the complex (*S. baltica*, *S. scandinavica*, and *S. vaxholmensis*). *S. hafniensis* (SP1S1-9) and *S*. *oncorhynchi* (L-17 and SP1S1-3), also isolated from sediments in the same study areas, were used as outgroups for comparison. Genomes in the heatmap are ordered according to ANIb similarity.

We reconstructed the evolutionary history of *S. baltica* complex strains. Our Bayesian inference based on the core proteome of the complete reference genomes (n = 32), revealed that an ancestral lineage split into two main branches, one leading to G1 and the other leading to G2 and G3 (Fig 6A). To better understand the mechanisms of this genomic diversification, we investigated whether homologous recombination plays a major role in this process, focusing on *S. baltica* strains recovered exclusively from sediments (n = 94). To this end, we applied two complementary approaches: (i) Gubbins, which identifies regions of elevated SNP density across the core genome alignment to detect recombination hotspots [39], and (ii) a recently described method that compares the number of identical genes (>99.8% nucleotide sequence identity) between genome pairs (observed F100) against the number expected by chance from FastANI-based relatedness, enabling inference of recent recombination frequency and gene flow within and between groups while accounting for shared ancestry [40].

First, to characterize the recombination dynamics within each group, we used Gubbins to compute different metrics, i.e., the ratio of recombination events to point mutations (*ρ/θ*; Fig 6B), the relative impact of recombination versus mutation on accumulated variation (*r/m*; Fig 6C) and mean of the recombinant fragment length (Fig 6D). G1 showed the lowest values across all metrics (*ρ/θ* = 0.008, *r/m* = 0.155, recombinant fragment length = 174 ± 139 bp), indicating that mutation dominates sequence diversification in this lineage. In contrast, G2 and G3 displayed substantially higher ratios and longer recombination fragments (*ρ/θ* = 0.162-0.267, *r/m* = 6.6-11.7, and recombinant fragment length = 658–734 bp), demonstrating a far greater contribution of recombination to genomic diversity (Fig 6B-6D).

Second, we performed pairwise genome comparisons using the F100-based approach, which yielded results consistent with those obtained using Gubbins (Fig 6E, 6F). As expected, F100 values dropped sharply between *S. baltica* complex members and more distant species isolated from Vaxön sediments, e.g., *S. hafniensis* (strain SP1S2-9) and *S. oncorhynchi* (strains L-17 and SP1S1-3), sharing less than 1% of genes [ln(F100) > 4.5] (Fig 6B). However, we observed high levels of gene sharing within the *S. baltica* complex strains (median 3.94; Fig 6E, 6F). These values are comparable to those observed in highly cohesive species with FastANI > 97%, both inside and outside the genus *Shewanella*, such as *S. algae* or *Salinibacter ruber* [41] (S7A, S7B Fig). Gene sharing was lowest in G1 (2.73 ± 0.51%) and highest in G3 (13.6 ± 9.84%), with G2 showing intermediate values (5.95 ± 2.28%). Between-group sharing was markedly higher between G2 and G3 (5.3 ± 1.32%) than between G1 and either group (~1.95 ± 1.49%), pointing to recent gene flow predominantly among the more recombinogenic lineages. Importantly, our F100 approach shows much higher gene sharing within species or genospecies than between them, supporting their delineation as discrete genomic units. To quantify this further, we counted genes classified as recently recombined across all pairwise comparisons, identifying 12,326 recombination events (62% core, 38% accessory genes), distributed across the entire genome (S8A Fig). After excluding genome pairs with ANIb > 98% (1.2% of all pairwise comparisons) to avoid overestimating recombination from very closely related strains, which exhibited high recombination levels (>45%, S8B Fig), average pairwise recombination was ~ 1.6 ± 0.5% within G1, increasing to ~4.9 ± 0.6% within G2, ~ 4.6 ± 2.3% within G3, and ~3.8 ± 1.6% between G2 and G3, with all values significantly higher than those involving G1 (*p* < 0.05; Fig 6G). These results indicate that G1, despite being mostly a single species (*S. baltica*), exhibits less gene sharing than the multi-species groups G2 and G3, reflecting extensive gene flow within the *S. baltica* complex, particularly between G2 and G3.

We next used F100 to estimate the *r/m* ratio between genome pairs and investigate recombination dynamics within the groups (Fig 6H). Within G2 and G3, strains forming part of the same species or genospecies exhibited most of the *r/m* ratios ranging from 1 to 100. Overall, the F100 estimates showed similar trends as Gubbins inferences, with G1 showing much lower *r/m* ratios than G2 and G3, in the range of 0.05 and 0.2, suggesting that point mutations predominantly drive diversification in this group. In G2 and G3, most *r/m* values ranged from 0.2 to 0.8 across all pairwise comparisons, showing that the divergence of these groups could be driven by ‘sexual’ speciation.

### The *S. baltica* complex is under purifying selection

Finally, we quantified selective pressures in single-copy core genes across G1, G2, and G3 (S9A, S9B Fig). The mean *dN/dS* (ω) across all groups was 0.08 ± 0.11, and approximately 99.5% of core genes exhibited ω ≪ 1 (S9A Fig), indicating strong evolutionary constraint. Significant variation in mean ω was observed among groups (*p* < 0.05, S9A Fig). The mean ω of G3 was approximately 1.5-fold higher than that of G1 and G2, with values of 0.11, 0.08, and 0.06, respectively, and G3 contained the largest number of genes with ω > 0.5, including up to 25 core genes, and a subset of six genes inferred to be under positive selection (ω > 1) (S9B Fig), all annotated as hypothetical protein-coding genes. The overall distribution of low ω values across groups is consistent with genome-wide patterns observed in other bacterial populations [42], showing that the evolutionary dynamics within the *S. baltica* complex is dominated by strong purifying selection.

## Discussion

What constitutes a bacterial species and the processes underlying bacterial speciation remain as open questions that are often challenging to address empirically in natural populations. Speciation is generally understood as the emergence of genomically cohesive lineages that evolve together while gradually becoming reproductively isolated through barriers to gene flow [7,14]. However, such isolation is frequently incomplete, as distinct bacterial species can maintain a certain level of gene exchange through recombination [43]. Numerous models have been proposed to describe the multifaceted modes of speciation in bacteria, which can enormously vary depending on biological and ecological factors [20,44–47]. The *S. baltica* complex provides a direct empirical demonstration of species concepts that view species as metapopulation lineages shaped by genetic exchange and shared ecological pressures along a continuous speciation spectrum [18,21]. Within this complex, coexisting, yet genomically differentiated lineages incipiently simpatrically diverge in the sediments, driven by homologous recombination and ecological specialization.

In this study, we identified closely related populations comprising three distinct evolutionary and functional groups, referred to as G1, G2, and G3, respectively. Our analysis of genomic relatedness in members of the *S*. *baltica* complex, as inferred by all-against-all ANIb determinations, revealed a bimodal distribution. One major group of ANIb interactions, ranging from 95.6% to 97.5% mainly involved *S. baltica* strains found in both the water column and the sediment. Remarkably, a second group of ANIb interactions (94.5–95.6%) comprised predominantly G2 and G3 members, encompassing numerous sediment-native genomic species according to current taxonomic delineation criteria [34], i.e., three species with standing in nomenclature, which we had previously described [32,33], and 11 putative genospecies identified in this study. Such an ANIb distribution is remarkable, as the ANIb range preponderantly comprising interactions involving G2 and G3 members is usually scarcely populated and represents a so-called ‘gray zone’, that is, a gap separating distinct species [4,48].

The analysis of G1 evolution primarily highlights *cladogenesis* at the subspecific level. This distinction is important because *cladogenesis* and *speciation* are not intrinsically synonymous. Originally introduced by B. Rensch, ‘cladogenesis’ refers broadly to phylogenetic branching and splitting [49,50]. In prokaryotes, a cladogenetic event does not necessarily result in the formation of two distinct gene pools that qualify as separate biological species, and it might instead produce subspecific units such as genomovars, phylogroups, or ecological subspecies. Examples include the diverse G1 ecotypes examined in this study, as well as those discussed in previous taxonomic revisions [51,52], which may or may not coexist indefinitely with the parental lineage or experience ongoing gene flow with it. The *S. baltica* complex is notable because the genomic relatedness of its members lies near the threshold for prokaryotic species circumscription, making cladogenetic events more likely to lead to full speciation rather than mere subspecific divergence. This is illustrated in several G1 lineages that cannot be classified as coherent genospecies, and particularly in G2 and G3 members, which share a common ancestor that already diverged from the *S. baltica* lineage, as shown in our Bayesian phylogenetic reconstruction.

Speciation involves genomic differentiation and a consequent reduction in recombination between emerging lineages. The ‘porous’ nature of gene flow barriers in bacteria means that incipient species can retain substantial recombination with related lineages [53], a phenomenon we observed within the *S. baltica* complex. While recombination within the same species (or genospecies) of G2 and G3 exceeded 45%, this value dropped sharply between different lineages. Of note, we still observed sustained homologous recombination between different lineages of G2 and G3 (5–7%), compared to less than 1% with G1 strains. This gene flow is insufficient to homogenize their genomic differentiation (which would therefore abrogate speciation) and rather points to an ongoing process of sympatric speciation. This is further supported by the temporal stability of several divergent genotypes. The repeated recovery of the same species and genospecies in both 2021 and 2023, along with their high levels of recombination indicates that these divergent strain groups represent stable, independently evolving lineages. Evolutionary models suggest that early-stage sympatric recombination can facilitate niche adaptation by combining beneficial alleles, whereas at later stages, recombination can produce low-fitness intermediates and counteract divergence [16,54,55]. Such trade-offs can be resolved by reducing the number of loci under selection or by progressively restricting recombination [54], being the onset of the latter process likely observed between G2 and G3 members.

The speciation process experienced by G2 and G3 members is further supported by genomic and physiological signatures of distinct ecological specialization, which plays an important role in bacterial diversification [56]. The predominance of genospecies emerging from sediments suggests that a sediment-associated lifestyle promotes evolutionary divergence. In surficial sediments, local variations in nutrient availability, oxygen concentration, or anaerobic electron acceptors, along with other physicochemical or biological factors, can create microenvironments favoring specific adaptations [57]. Such fine-scale heterogeneity might remain undetected by metagenomic inference, as could be the case in this study. Regardless, rapid metabolic divergence can occur even in laboratory-recreated homogeneous environments, increasing intraspecies heterogeneity [45,58]. Beyond the test tube, evidence shows that genomic and gene expression variations promote ecological divergence of closely related bacteria in natural ecosystems [59].

In the *S*. *baltica* complex, several genomic features may contribute to ecological differentiation among groups. Genomic enrichment in metabolic resource-utilization pathways differentiates the strain groups, suggesting distinct ecological strategies among G1, G2, and G3 members in sediments. This was reflected in our carbon substrate utilization assays, where G2 strains showed more proficient utilization of organic compounds such as *N*-acetyl-D-glucosamine. Ecological differentiation may also arise from variation in energy-acquisition pathways, which are widely gained or lost independently of isolation depth, as exemplified by dissimilatory sulfite reduction, a capacity that was highly conserved across G3 members (100%) as compared to G2 (56%) and G1 strains (21%). Sulfur compounds have supported microbial energy metabolism since the early stages of Earth’s history and remain important in aquatic sediments, where partially reduced inorganic sulfur compounds such as sulfite contribute to sulfur biogeochemical cycling and serve as energy sources for metabolically versatile *Shewanella* spp. [60,61]. Our phylogenetic and synteny analyses suggest that recombination has shaped the distribution of *sir* cassettes within the *S*. *baltica* complex. The presence of two distinct *sir* cassette types in G1 vs. G2/G3, each more closely related to cassettes found in other *Shewanella* species (*S*. *oncorhynchi* and *S*. *xiamenensis*, respectively) than to one another, is consistent with lineage-specific acquisition and subsequent divergence of these loci during the diversification of the *S*. *baltica* complex. Notably, two Baltic Sea G1 strains (GCA_001620325.1 and GCA_000017325.1) carry a *sir* cassette whose SirA proteins cluster phylogenetically with those of G2 and G3 strains, indicating that recombination has also contributed to the redistribution of this locus across lineages. A similar pattern of diversification and interspecific exchange of genes involved in sulfur-compound respiration has been reported in *S*. *algae*, where DMSO respiration is variable among strains, including complete loss of the canonical *dmsEFABGH* operon in some isolates [62]. Conversely, divergent DmsA homologs in strains A56 and CCUG 58400 were found to be closely related to the DmsA of *S*. *chilikensis*, suggesting acquisition of DMSO-respiratory machinery from another *Shewanella* lineage [62]. Together, these observations point to sulfur compound-respiration loci as particularly dynamic regions of *Shewanella* genomes, shaped by recurrent gene gain, loss, and recombination. Consistent with this broader pattern, respiratory traits involving sulfur compounds are highly variable within the *S*. *baltica* complex, with tetrathionate reduction absent from some G1 members and DMSO reduction variably distributed across G1-G3. Our genomic and experimental data are therefore consistent with ecological resource specialization within a shared ecosystem, particularly in the use of sulfur compounds, as one plausible driver of ecological differentiation among G1-G3, rather than differentiation driven primarily by spatial isolation or redox stratification. Beyond these metabolic differences, the substantial pool of plasmid-borne genes of unknown function may provide an additional, yet unresolved, ecological toolset for their hosts, although their contribution to ecological differentiation or speciation remains unclear.

**Fig 7:**
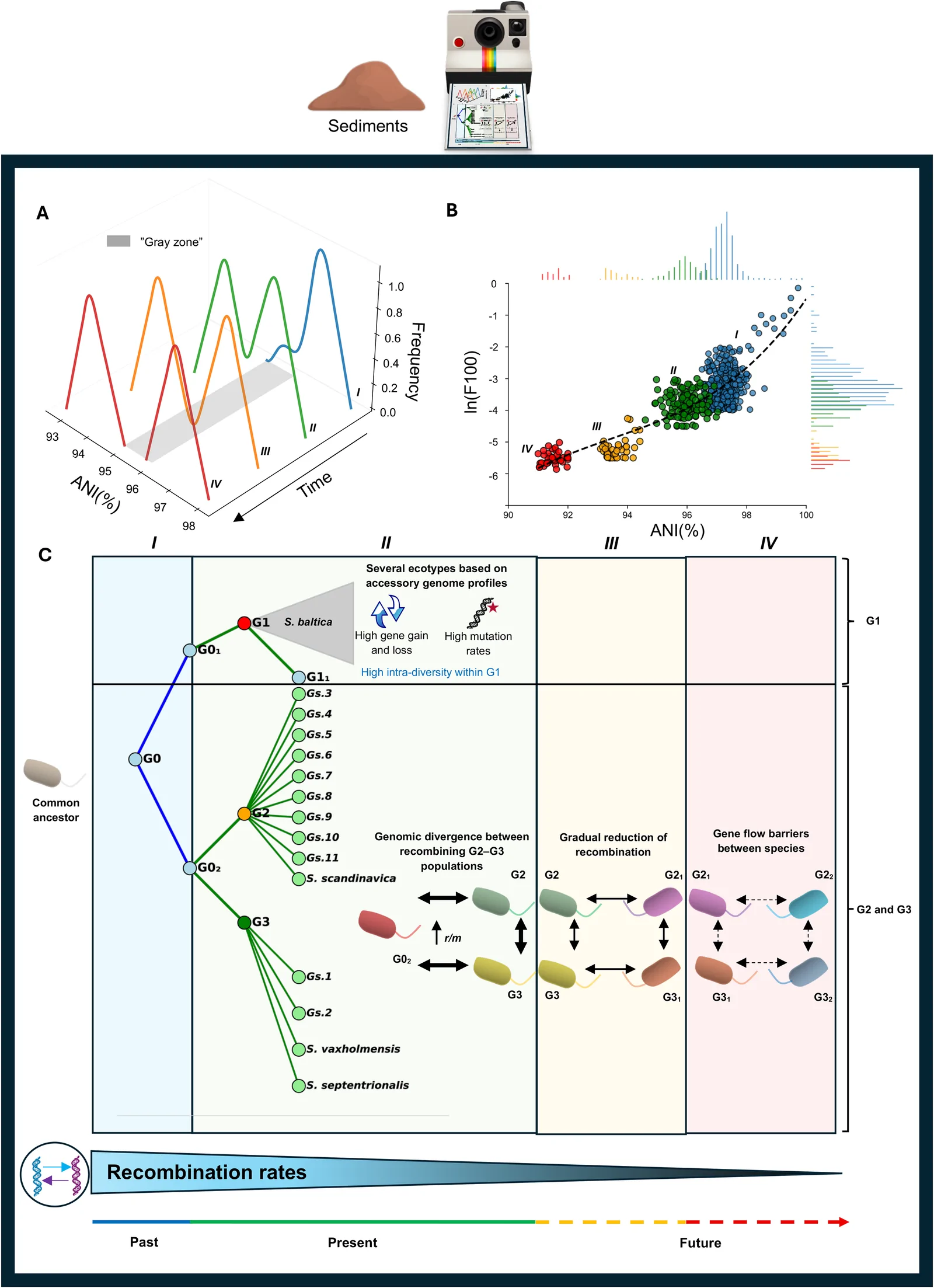
Speciation model for the *S*. *baltica* complex. **(A)** Conceptual model for the *S. baltica* complex adapted from complex from Achtman and Wagner [20]. **(B)** Recombination dynamics within the complex. **(C)** Evolutionary trajectories within the *S. baltica* complex. These models show how lineages progressively diverge through four phases from a common ancestor. In phase I (blue), high recombination maintains genomic cohesion within the ancestral lineage (G0), while subpopulations gradually emerge and begin evolving independently (G01 and G02). In phase II (green), recombination rates decline but remain substantial. During this stage, G01 gave rise to the G1 population (predominantly *S. baltica*), characterized by low *r/m* ratios and high gene turnover, whereas G02 differentiated into the G2 and G3 lineages, representing incipient, independently evolving species (genospecies) whose divergence is likely driven by homologous recombination is likely driven by homologous recombination and ecological differentiation. In phase III (orange), recombination decreases further, resulting in increasing genomic isolation between lineages. In phase IV (red), lineages become fully differentiated species with minimal recombination and effective genomic isolation. Figures were generated using custom Python scripts integrating ANI values, recombination dynamics, and phylogenetic tree visualizations. Icons were obtained from https://openclipart.org/ and https://pixabay.com/.

Collectively, these observations place the *S. baltica* complex along a continuum of lineage divergence consistent with lineage-based models of bacterial speciation, such as de Queiroz’s method-free concept [18] (and related work by Hey [19]), its subsequent adaptation by Achtman and Wagner [20], and the speciation-spectrum model of Shapiro and Polz [21,63]. In these models, populations do not form discrete, fully isolated units but behave as metapopulation lineages that maintain internal genomic cohesion while gradually reducing gene flow. Thus, species correspond to independently evolving lineages and are not necessarily phenotypically distinguishable or ecologically isolated [20]. Divergence therefore proceeds along a continuum, from (i) emergence of distinct intraspecific sub-clades, to (ii) incipiently divergent lineages, to (iii) partially genomic differentiated species linked by genome intermediates, and ultimately to (iv) fully resolve species. Applying this model to the *S. baltica* complex, we infer that an ancestral population (G0) split into two subpopulations, G01 and G02, which subsequently followed distinct evolutionary trajectories (phase I). G01 gave rise to the present-day G1 population, largely dominated by *S. baltica* and characterized by low *r/m* ratios together with elevated gene gain and loss, generating extensive genomic and metabolic diversity. In contrast, G02 diversified into the G2 and G3 lineages that remain internally cohesive yet increasingly differentiated from one another (phase II). Their persistence over multiple years (meaning successful ecological adaptation) and ongoing homologous recombination, indicates a metapopulation structure in which divergence is actively occurring. As recombination declines between G2 and G3, genomic isolation increases and divergence continues, generating genomic intermediates (phase III). Notably, *S*. *septentrionalis*, despite belonging to the *S*. *baltica* complex as a G3 representative, appears to be more closely related to *S*. *hafniensis* than to *S*. *baltica*, consistent with a possible placement of *S. septentrionalis* in a more advanced stage of the speciation continuum (phase III). Finally, continued divergence and a further decline in recombination could give rise to fully genomically isolated species (phase IV), with *S. oncorhynchi* as a putative example. The proposed model is represented in Fig 7A-7C.

Together, our findings capture a snapshot of sympatric speciation, revealing the coexistence of incipient yet genomically differentiated lineages within a shared microhabitat lacking obvious spatial barriers. The extent of diversity uncovered in the sediment populations analyzed here has not previously been observed among *S. baltica* strains isolated from the stratified Baltic Sea water column [28,29] or from any other environments, pointing to a sediment-associated lifestyle as a potential catalyst for the emergence of novel *Shewanella* genotypes through recombination-driven (‘sexual’) speciation and metabolic specialization. Confinement, increased cell-to-cell interactions, or the existence of a diversity of micro-niches within the sediment might actively contribute to this process. Support for the view of sediments as ‘diversification hotspots’ in *Shewanella* is provided by the documented coexistence of multiple recently described *Shewanella* species in mangrove surficial sediments, whose close genomic relatedness to *S. loihica* mirrors the diversification patterns observed within the *S. baltica* complex [64]. Notably, the evidence presented here extend the known limits of sympatric speciation beyond the formation of subspecific ecotypes by demonstrating that bacterial species can diverge and maintain distinct lineages despite ongoing recombination, without spatial segregation and at very fine spatial scales. Collective interactions and metabolic, and therefore, ecological, differentiation in sediments may structure bacterial populations strongly enough to promote divergence at the species level. Future work should determine how sediment microhabitats function as crucibles for speciation, delve into the mechanisms driving divergence under continued gene flow, and identify the precise ecological determinants that sustain the coexistence and persistence of emerging genotypes.

## Materials and methods

### Sample collection, environmental DNA extraction, and bacterial isolation

Between 2021 and 2023, surficial sediment cores were aseptically collected at Vaxön (Stockholm archipelago, 59°24′01.0″ N 18°20′20.2″ E) using sterile 30 ml syringes (VWR Cat. No. 613–2035). Samples were transported to the laboratory within 2 h, and aliquots for DNA extraction and bacterial isolation were taken at 0, 2, 4, and 6 cm depth. DNA (ca. 0.5-0.8 mg) was extracted using the DNeasy PowerSoil Pro kit (Qiagen, Hilden, Germany), after mechanical disruption with FastPrep-24 5G (MP Biomedicals; 4 × 40 s at 5.5 m/s) and stored at -80 °C until sequencing. For bacterial isolation, sediment aliquots were suspended in phosphate-buffered saline (0.01 M, pH 7.4), vortexed, serially diluted, and plated (100 µl) on Lyngby’s Iron Agar (LIA) with 0.04% (w/v) L-cysteine. Plates were incubated at 28 °C for 24 h. *Shewanella*-like colonies, characterized by black centers with pale orangish margins, were re-streaked three times on LIA and identified to the genus level by MALDI-TOF (MALDI Biotyper Sirius, Bruker). Additional sediment and water samples were collected from Hagaparken (59.3546 N, 18.0429 E), Lidingö (59.3667 N, 18.1757 E), Nynäshamn (58.8995 N, 17.9508 E), and Tyresö (59.2324 N, 18.3110 E) between 2021 and 2022 (S1 Fig), and processed as previously described [32,33]. In total, 112 *Shewanella* strains were isolated and analyzed, including previously reported strains [32,33].

### Whole-genome and metagenome DNA sequencing

Genomic DNA was extracted from overnight LB (Miller) cultures at 28 ºC using the DNeasy Blood & Tissue kit (Qiagen) and quantified with the Quant-iT High Sensitivity dsDNA assay (ThermoFisher, Waltham, MA, USA). Equimolarly pooled libraries were circularized using the MGI Easy Circularization kit, and 2 × 100 bp paired-end sequencing was performed on a DNBSEQ-G400 instrument (MGI, Shenzhen, China). Genome assemblies were generated with SPAdes v3.15.5 [65] via Bactopia v1.7.1 [66] or BACTpipe (https://github.com/ctmrbio/BACTpipe). For 32 representative strains, including type strains and at least one novel genospecies, Oxford Nanopore sequencing was performed to obtain complete genome sequences. High-molecular-weight DNA was extracted using the Quick-DNA HMW MagBead Kit (Zymo Research, Irvine, CA, USA), and libraries were prepared with the Rapid Barcoding Kit (SQK-RBK114.24). Sequencing was carried out on a FLO-MIN114 (R10.4.1) flow cell using a MinION Mk1B device (Oxford Nanopore Technologies), using MinKNOW v25.05.14 with high-accuracy basecalling modelling. Long reads were assembled *de novo* using Flye v2.9.5-b1801 [67] and Raven v1.8.3 [68]. Hybrid assemblies combining Oxford Nanopore long reads and DNBSEQ-G400 reads were generated using Unicycler v0.5.1 to optimize plasmid recovery [69,70]. Circularized replicons produced by Flye were retained whenever possible.The resulting genome sequences were polished using Medaka v1.11.3 (https://github.com/nanoporetech/medaka), Polypolish v0.6.0 [71], and POLCA within MaSuRCA v4.1.0 [72,73], and reordered using UGENE v48.1 [74] based on the Unicycler start genes database [70,74]. Assembly quality metrics were evaluated using QUAST [75] v5.0.2. Genome completeness and contamination were evaluated using CheckM [76] and ConFindr [77]. The genomes were of high quality, exhibiting an average of 6 ± 4 single nucleotide variants (SNVs) in core genes, with mean completeness of 98.76 ± 0.86% and contamination of 0.74 ± 0.63%, indicating a low likelihood of chimeric assemblies (S2 Table).

For metagenomic analysis, 2 × 150 bp paired-end sequencing was performed on a DNBSEQ-T7 (MGI). Quality-trimmed reads were classified with Kraken2 v2.1.0 [78] using the Genome Taxonomy Database (GTDB, downloaded 8 April 2022) [79]. Contigs were assembled with MEGAHIT v1.2.9 [80] and analyzed with the SqueezeMeta v1.7.2 pipeline [81]. Open reading frames (ORFs) were predicted with Prodigal v2.6.3 [82], and protein similarity searches were performed against the GenBank non-redundant (nr) protein and Kyoto Encyclopedia of Genes and Genome (KEGG) databases using DIAMOND (v2.1.10) [83]. Finally, reads were mapped back to contigs with Bowtie2 v2.5.4 [84] to assess functional coverage and abundance.

### Genomic relatedness analyses

Average nucleotide identity (ANI) analyses were performed using JSpeciesWS for BLAST-based ANI (ANIb) [85] and FastANI v1.34 (‘many to many’ mode, default settings; https://github.com/ParBLiSS/FastANI). Pairwise ANIb values were hierarchically clustered to define genome groups. Publicly available genome sequences of *Shewanella algae* (n = 219), *S. xiamenensis* (n = 98), and *S. oncorhynchi* (n = 32, including three strains from this study) were downloaded from NCBI GenBank on 31 May 2025 and analyzed with FastANI. Digital DNA–DNA hybridization (dDDH) values were calculated using the Genome-to-Genome Distance Calculator (GGDC) [86], comparing each genome against the maximum allowed number (n = 75), and repeated for all genomes. Only results from the recommended Formula 2 were reported [87].

### Pangenome analyses, accessory genome clustering and gene gain-loss dynamics

Pangenome analyses within the *S. baltica* complex (and the additional datasets previously mentioned) were performed on genomes annotated with Prokka v1.14.6 [88], using PPanGGOLiN v2.2 [89]. Default parameters were applied with ≥90% identity and coverage, as higher thresholds (>95%) over-split conserved genes and lower thresholds (50–80%) merged paralogs. Genes present in ≥95% of genomes were classified as core, considering that the vast majority of the genomes sequences are draft genomes. Core gene and protein family alignments were generated using fast Fourier transform (MAFFT) [90], and phylogenomic trees were reconstructed with FastTree v2.1.10 [91] using GTR + Γ4 for DNA alignments and JTT + CAT for protein alignments. Trees were bootstrap-supported with 1,000 replicates, midpoint-rooted, and visualized in iTOL v6 6 [92]. Accessory gene accumulation curves were built from the Rtab presence-absence matrix by randomly permuting genome order 100 times and averaging the cumulative number of new accessory genes at each step. Heaps’ law (n = k·N^γ^) was fitted to the mean curve by non-linear least-squares regression, with γ indicating pangenome openness (closer to 0 = closed, closer to 1 = open). Using the same matrix, we constructed a hierarchical clustering dendrogram based on gene presence–absence. This matrix was also analyzed with Mandrake v1.2.2 to generate a two-dimensional embedding [93], enabling identification of accessory genome clusters (AGCs). Gene gain and loss events were modeled using Panstripe v0.3.1, using the included Gaussian distribution, which provides more robust model fitting while yielding results comparable to the compound poisson (Tweedie) model [36]. Using PanRGP as implemented in PPanGGoLIN, we identified regions of genome plasticity (RGPs), defined by PanRGP as clusters of consecutive shell or cloud genes [94], which was used as a proxy for horizontal gene transfer (HGT).

### Estimation of genome-wide differentiation

Genome assemblies were aligned with Split K-mer Analysis V2 using *S. baltica* CECT 323^T^ as the reference, and single nucleotide polymorphisms (SNPs) were extracted with SNP-sites v2.5.1 [95] and stored in VCF format. SNPs were grouped into overlapping 10 kb sliding windows (2.5 kb steps), and for each window, pairwise differentiation was calculated as the proportion of discordant SNPs (*p*-distance). Genome-wide differentiation between each pair of genomes was obtained as the average *p*-distance across all windows, providing a genome-wide complement to classical population-genetic metrics ranging from 0 (identical genomes) to 1 (completely different) [96]. Pairwise distances were concatenated and clustered as for ANIb.

### Bayesian phylogenetic analysis

Bayesian phylogenetic analysis was performed with BEAST X v10.5.0 [97] using the core proteome alignment of 32 complete genomes (3,396 proteins; 1,157,272 amino acids) representing distinct species and putative genospecies within the  *S. baltica* complex. A Blosum62 substitution model was applied, with a strict molecular clock to assume uniform substitution rates, and no time calibration was used, as the aim was to assess relative relationships rather than absolute divergence times [98,99]. The starting tree was generated under a constant-size coalescent, and a Yule speciation model constrained tree topology and branch lengths. Posterior distributions were estimated via Markov Chain Monte Carlo (MCMC) for 10,000,000 generations, sampling every 1,000 steps. Convergence was assessed with Tracer v1.7.2 [97], trees were summarized with TreeAnnotator v10.5.0 [97], and visualized using iTOL v6 [92].

### Metabolic and horizontally acquired gene profiling for ecological differentiation

Genome assemblies were re-annotated using COGclassifier v1.0.5, based on COG categories (https://github.com/moshi4/COGclassifier/tree/main), and RAST SEED roles [100], by calculating the proportion of protein-coding genes assigned to each functional category (performed in KBase [101]). Taxonomic assignments for the COGs identified within RGPs were obtained using EggNOG v5.0 [102]. Functional enrichment was assessed using Kruskal-Wallis tests for COG categories and SEED roles, and fold changes were calculated as the median value per genome relative to all others. COG and RAST-annotated genomes, and COG-annotated HGT genes, were compiled into feature matrices and standardized to Z-scores prior to multivariate analyses. Group differences were assessed using permutation-based multivariate analysis of variance (PERMANOVA), and enrichment analyses used Benjamini–Hochberg false discovery rate (FDR) correction, with significance defined as FDR-adjusted *p* < 0.05. Finally, KEGG pathway module completeness was evaluated from KEGG orthologues (KOs) presence-absence using the kegg-pathways-completeness tool (https://github.com/EBI-Metagenomics/kegg-pathways-completeness-tool/tree/master).

Respiratory metabolism of *N*- and *S*-oxides was investigated using annotated sequences of respiratory reductases from reference species within the complex, including *S. baltica* OS195 (GCA_000018765.1), *S. baltica* CECT 323^T^ (GCA_900456975.1), and *S. scandinavica* SP2S1-2^T^ (GCA_031932065.1). These genomes contained reductases for nitrate (NapA; ABX48036, ABN61416, MDT3282522, MDT3279942, MEM6250911, MEM6249282), nitrite (NrfA; ABX50994, MDT3279623, MEM6250231.1), trimethylamine *N*-oxide (TMAO, TorA; ABX50514, MDT3281430.1, MEM6250556.1), dimethyl sulfoxide (DMSO, DmsA; ABX49397.1, MDT3282918.1), tetrathionate (TtrA; WP_006080095, MDT3279598.1, WP_311905661), thiosulfate (PhsA; MDT3282209.1, WP_311905956), and sulfite (SirA; ABX51162, MDT3279461, MEM6249422). After identifying homologs in our dataset, we constructed a custom database of these respiratory reductases designed to capture the diversity of evolutionary lineages within the complex. For the final analysis, the curated reference sequences from this database were used for BLASTp (BLAST+ v2.1.16) [103] searches with an e-value cutoff of 10^-30^, and hits with ≥90% sequence identity were retained for downstream analyses. Synteny analysis of the genomic regions containing the *sirA* gene was performed using Clinker [104]. SirA protein sequence alignments were performed using Clustal Omega, and phylogenetic trees were reconstructed with MEGA12 using the maximum-likelihood method and the best-fit amino acid substitution model identified by the software (WAG+I), with 1,000 bootstrap replications.

### Experimental metabolic characterization

The ability to reduce sulfite of G1, G2 and G3 members was assessed with an H₂S detection agar medium, consisting of 1% (w/v) basal agar medium containing 7.5 g/L NaCl, 5 g/L MgCl_2_·6H_2_O, 0.5 g/L KCl, 0.15 g/L CaCl_2_·2H_2_O, 1 g/L NH_4_Cl, and 0.2 g/L KH_2_PO_4_, supplemented with 0.02% (w/v) casamino acids, 0.015% (w/v) FeSO_4_, 40 mM sodium lactate, and 10 mM sodium sulfite. The medium was buffered with 30 mM HEPES and adjusted to pH 7.6. After 48 h of incubation at 28 ºC, the formation of black precipitates was considered a positive reaction.

In addition, carbon source utilization across representative strains of each group was evaluated using Biolog EcoPlates (G1, n = 18; G2, n = 23; and G3, n = 12). Prior to the analysis, strains were grown overnight in Miller’s LB broth, diluted 1:100, and cultured until reaching the exponential phase (ca. 0.6 OD_600_). Bacteria were then pelleted, resuspended in 0.85% (w/v) NaCl, and cell densities normalized to McFarland 0.5 before transferring 150 µL to each well. Plates were incubated for 72 h, with OD_590_ monitored every 12 h.

### Detection of recombination events

Homologous recombination was measured using Gubbins [39], and the F100 prokaryotic recombination pipeline [40] (https://github.com/rotheconrad/F100_Prok_Recombination). Using the core genome alignment as input, we ran Gubbins separately for each of the three evolutionary groups identified in this study, applying default parameters (minimum number of SNPs to identify a recombination block = 3; minimum window size = 100 bp; maximum window size = 10,000 bp). For the F100 pipeline, reciprocal best matches (RBMs) between genome pairs were identified by BLAST [105], and the F100 metric was calculated as the proportion of RBMs that were 100% identical, serving as an indicator of recent gene exchange. Recombinant genes were classified as core (≥90% of genomes), accessory (<90% and present in multiple genomes), or genome-specific, while highly conserved genes (as the 10% of core genes with the lowest divergence) were excluded to avoid conflating conservation with recombination. Gene clustering was performed with MMSeqs2 using the same parameters as for core gene definition (--min-seq-id 0.90, -c 0.90, --cov-mode 1, --cluster-mode 2, --cluster-reassign), and pairwise genome recombination values were extracted from the pipeline output (script *03f_Recombinant_pair_analysis.py*, default settings). Additional recombination parameters were calculated as the recombination-to-mutation ratio (*ρ/θ*) or the relative impact of recombination versus mutation on accumulated variation (*r/m*) within the groups was calculated using Gubbins pipeline. Pairwise *r/m* estimates were obtained using the empirical approach implemented in the F100 prokaryotic recombination pipeline [40], which assumes that recombined genes initially carried the average sequence divergence of the compared genomes.

### Evolutionary genomic analyses

Selection acting on core genes of the *S. baltica* complex was assessed using codon alignments of single-copy core genes for each respective phylogenetic group [106]. Protein alignments were back-translated to codon alignments using PAL2NAL v14 to preserve reading frame information. Gene-specific phylogenies were reconstructed with FastTree v2.1.11, and branch-specific ratios of nonsynonymous to synonymous substitution rates (*dN/dS,* ω) were estimated using the FitMG94 model, which implements the Muse–Gaut (MG94) codon substitution model of sequence evolution, in HyPhy v2.5.64 [107]. The model was implemented to permit independent variation of *dN* and *dS* across branches, thereby accommodating lineage-specific heterogeneity in selective pressures. Confidence intervals for ω were estimated using profile likelihood, and deviations from neutrality (ω = 1) were evaluated using likelihood ratio tests comparing the unconstrained model to a nested model in which ω was fixed at 1. To ensure robust downstream inference, HyPhy outputs were filtered to retain genes with codon alignments of at least 60 codons, and estimates were excluded when *dS* were below 0.005 to minimize instability and avoid artificially inflated ω values associated with extremely small *dS* values. For core genes showing signatures of positive selection, we additionally screened for homologous recombination, and removed if it was the case, using the GARD method [108] implemented in HyPhy, since recombination can produce false signals of adaptive evolution by affecting *dN/dS* estimates.

### General computational methods, statistics, and data availability

Data processing, plotting, and statistical analyses were performed using custom Python (v3.9.19) and Bash (GNU Bash v3.2.57) scripts, together with RStudio v2025.05.1. All analysis scripts used in this study available via the Martín-Rodríguez Lab GitHub repository (https://github.com/Martin-Rodriguez-lab/Life_in_sediments_fosters_sexual_speciation_in_the_Shewanella_baltica_complex) and multiple sequence alignments and datasets used and/or analyzed to reproduce the analysis can be found in our Zenodo for this project (https://zenodo.org/records/21233620). Genome assemblies are available in NCBI under BioProject PRJNA526057 and metagenomic raw reads under SRR36813638-SRR36813649, with individual accession numbers listed in S2 Table.

## Supporting information

S1 Fig Geographic locations of sampling sites in the Stockholm region. The map shows the number of isolates collected at each site and used in this study (n = 112). The following abbreviations are used refer to the sampling sites; Växon 2023, VAX-SP (59.3999 N, 18.3374 E); Växon 2021, SP (59.401044 N, 18.325925 E); Hagaparken, H (59.355833 N, 18.043611 E); Lidingö, L (59.367700 N, 18.156943 E); Tyresö, T (59.232778 N, 18.311111 E); Nynäshamn, N (58.899247 N, 17.950619 E). The size of the circle denotes the number of isolates from each sampling site. Coastline and country-boundary data were generated using the Basemap toolkit for Matplotlib, based on the GSHHG (Global Self-consistent, Hierarchical, High-resolution Geography Database) and WDBII (CIA World DataBank II) datasets (https://www.soest.hawaii.edu/wessel/gshhg/).
(PDF)

S2 Fig Taxonomic and functional profiles of metagenomic samples collected from different sediment depths. **(A)** Top 15 most abundant phyla with the Shannon diversity, and **(B)** beta-diversity analysis using principal component analysis (PCA) to assess genus-level dissimilarity between the different depths in Växon sediments. Analyses were conducted on classified reads using Kraken2 and Bracken2 against the GTDB database downloaded on April 8, 2022. **(C)** PCA and **(D)** heatmap of gene abundance per million (GPM) annotated contigs, derived from KEGG annotations.**(E)** Top 100 most abundant KEGG annotations-based GPM, categorized by functional class found across all the collected depths, with average values log_10_-transformed. The assembled contigs were KEGG annotated using the SqueezeMeta pipeline. **(F)** Relative abundance of *Shewanella* in the metagenomic bacterial reads.
(PDF)

S3 Fig Genomic diversity of the *S*. *baltica* complex based on ANI and shared genome fraction. **(A)** Frequency distributions of pairwise ANIb comparisons (0.1% windows) for *S. baltica* complex genomes (n = 109), including *S. hafniensis* (strain SP1S1-9 and 3 additional genome sequences from NCBI GenBank, S3 Table) and *S. baltica* CECT 323^T^ (top red histogram)*,* in combination with the shared gene content (blue dotplot). **(B)** Kernel density distribution of FastANI values, with distinct peaks corresponding to *S. baltica* (blue; genomes annotated as *S. baltica*, n = 70 in this study plus publicly available genomes annotated as *S. baltica,* n = 66), *S. xiamenensis* (green), *S. oncorhynchi* (yellow), and *S. algae* (red). All genomes were downloaded from the NCBI GenBank on 31 May 2025 (S3 Table).
(PDF)

S4 Fig Core genome-based and core proteome-based phylogenomic reconstructions of the *S. baltica* complex. **(A)** Combined core genome and proteome tree constructed using a 90% identity and 90% coverage threshold based 3,308 core gene and protein families from the 109 genomes analyzed. The type strain *S. baltica* CECT 323^T^ was included as a reference. Blue labels indicate genomes isolated from the water column. Within G2 and G3, additional colors indicate distinct species previously described, namely *S. scandinavica*, *S. vaxholmensis*, and *S. septentrionalis*, as well as genospecies (Gs.) identified in this study. The species delineation is explained in the main text. The numbers on the branches denote bootstrap support values after 1,000 replications. **(B)** Accumulation curves of accessory genome content of the *S. baltica* complex compared with the publicly available genomes of *S. xiamenensis* (green), *S. oncorhynchi* (yellow), and *S. algae* (red). All public genome sequences were downloaded from NCBI GenBank on 31 May 2025.
(PDF)

S5 Fig Plasmid characterization within the *S. baltica* complex. **(A)** Pairwise average FastANI comparison of all plasmids identified within the *S. baltica* complex among the 32 closed genomes (red = G1, orange = G2, and green = G3). Numbers after the name denote the plasmid number in that specific genome. **(B)** Cluster of orthologous groups (COG) annotation of the 21 detected plasmids. Detailed gene annotations are provided in S4 Table.
(PDF)

S6 Fig Completeness of KEGG metabolic pathways. KEGG pathways were annotated for the defined groups to assess the completeness of metabolic routes across the study strains within the *S. baltica* complex. Carbohydrate-related pathways, highlighted with arrows, were more complete in G1.
(PDF)

S7 Fig Genetic environment of *sir* cassettes in *S*. *baltica* complex strains and other *Shewanella* spp. Comparative gene synteny analysis of the genomic region around *sirA* (red boxes) reveals a variably distributed region of genome plasticity (RGP, cyan outline and enlarged above) comprising the inversely transcribed *sirABIGCDJKLM* and *sirEFG* operons and the transcriptional regulator *sirR*. Arrows represent coding sequences, with colors indicating homologous gene families. The degree of conservation of the represented genes across genome sequences is indicated by shades of gray and black in the connections. The presence transposons, which generally belonged to the IS10 family, is indicated by an orange frame.
(PDF)

S8 Fig F100 vs FastANI of (A) *S*. *algae* (n = 219) and (B) *Salinibacter ruber* (n = 102) from a saltern in Mallorca. The dashed black lines indicate the ln(F100) interaction boundaries between ln(F100) = -4 and ln(F100) = -3. For **(A)** and **(B)**, a Gam model using the public available genomes from NCBI (downloaded 31 May 2025) was built (r^2^ = 0.72), and turquoise colors denote genomes that do not follow the model.
(PDF)

S9 Fig Reciprocal best match (RBM) genes identified for representative *S. baltica* complex. **(A)** Pairwise RBM genes were identified across several genomes within the *S. baltica* complex, including VVAX-SP0-4CM-5 (G1), VAX-SP1-2CM-2 (G1), *S. scandinavica* SP2S1-2 (G2), SP1S1-7 (G2), *S. vaxholmensis* SP1S1-4 (G3), and S2S1-4 (G3). Each rectangular marker in the figure represents an individual gene, color-coded to distinguish highly conserved/universal genes (in red), core genes (in blue), and accessory genes (green). The vertical axis indicates nucleotide sequence identity of RBM genes shared between the seven query genomes (each shown as a separate row) and the reference genome (horizontal axis), where genes are ordered by their position in the reference genome. Green arrows indicate genomic islands unique to the reference genome, characterized by the absence of corresponding genes in other genomes in the same region. In contrast, red arrows point to highly conserved regions consistently shared within the pairwise comparisons. **(B)** Recombination comparison between genomes from different groups (G1, G2, and G3) of the *S. baltica* complex belonging to the same species, and ANIb > 98%. Asterisks indicate functional categories significantly enriched among recombinant genes, as determined by one-sided Chi-square tests (*p* < 0.05) with Benjamini-Hochberg correction, suggesting these functions might be preferentially transferred via homologous recombination.
(PDF)

S10 Fig Evolutionary genomic analyses. **(A)** Distribution of branch-specific *dN/dS* values across core genes in G1, G2, and G3. Boxplots show median and interquartile range, points represent individual genes, and error bars indicate standard deviation. **(B)** Mean *dN/dS* values per group across binned *dN/dS* 0.25 intervals (0-2.5). Bars represent group means within each window ± standard error, showing the number of core genes in each window for each group. In **(A)** and **(B)** the dashed line indicates neutrality (*dN/dS* = 1), and different letters denote significant differences among the groups (*p* < 0.05).
(PDF)

S1 Table Metagenomic summary and taxonomic composition of VAX sediment samples across sampling points (SP0, SP1, SP2, SP4) and depths (0–6 cm). For each sample, total raw reads, percentages of classified, unclassified, and microbial reads are reported. The relative abundance (%) of dominant bacterial genera is shown, along with the corresponding taxonomic lineage.
(XLSX)

S2 Table Metadata and genome assembly statistics for the isolates used in this study. Genomes were annotated using Prokka, and genome completeness and contamination were assessed with CheckM.
(XLSX)

S3 Table Public genome sequences from NCBI GenBank included in this study. Assembly accession numbers for representative strains of *S. baltica*, *S. algae*, S*. oncorhynchi*, and *S. xiamenensis* used for comparative genomic analyses are listed.
(XLSX)

S4 Table Plasmid annotations for study genomes. For each plasmid, the number and size (Kb), associated clade, coding sequences (CDSs), genomic location, and predicted gene product are listed.
(XLSX)

S5 Table Group-specific (exclusive) COGs. The table includes the COG identifier, functional category (COG letter), group assignment, and the strain carrying each COG.
(XLSX)

S6 Table Differentially enriched COG-annotated genes between groups. For each gene cluster, the COG identifier, functional category (COG letter), group assignment, fold change, and predicted gene product are reported.
(XLSX)

S7 Table Differentially abundant Rast-SEED functional roles identified between groups. For each role, the accessory group, comparison group, adjusted *p*-value, and fold change are reported.
(XLSX)

S8 Table Identification of reductases for alternative electron acceptors across study genomes. Query sequences were matched to group-specific reference proteins using sequence similarity searches, and alignment statistics (% identity, alignment length, mismatches), together with genome, group assignment, and sampling source, are reported.
(XLSX)

S9 Table Accession numbers for the SirA sequences used to construct the phylogenetic tree shown in Fig 5B. (XLSX)
