# Supplementary material for: Life in sediments fosters ‘sexual’ speciation in the Shewanella baltica complex
Source: PLoS Genet. 2026 Sep 11;22(9):e1012306. doi: 10.1371/journal.pgen.1012306 (PMC13600613; doi:10.1371/journal.pgen.1012306)
Supplement: Supplementary file 1 [file pgen.1012306.s001.pdf]

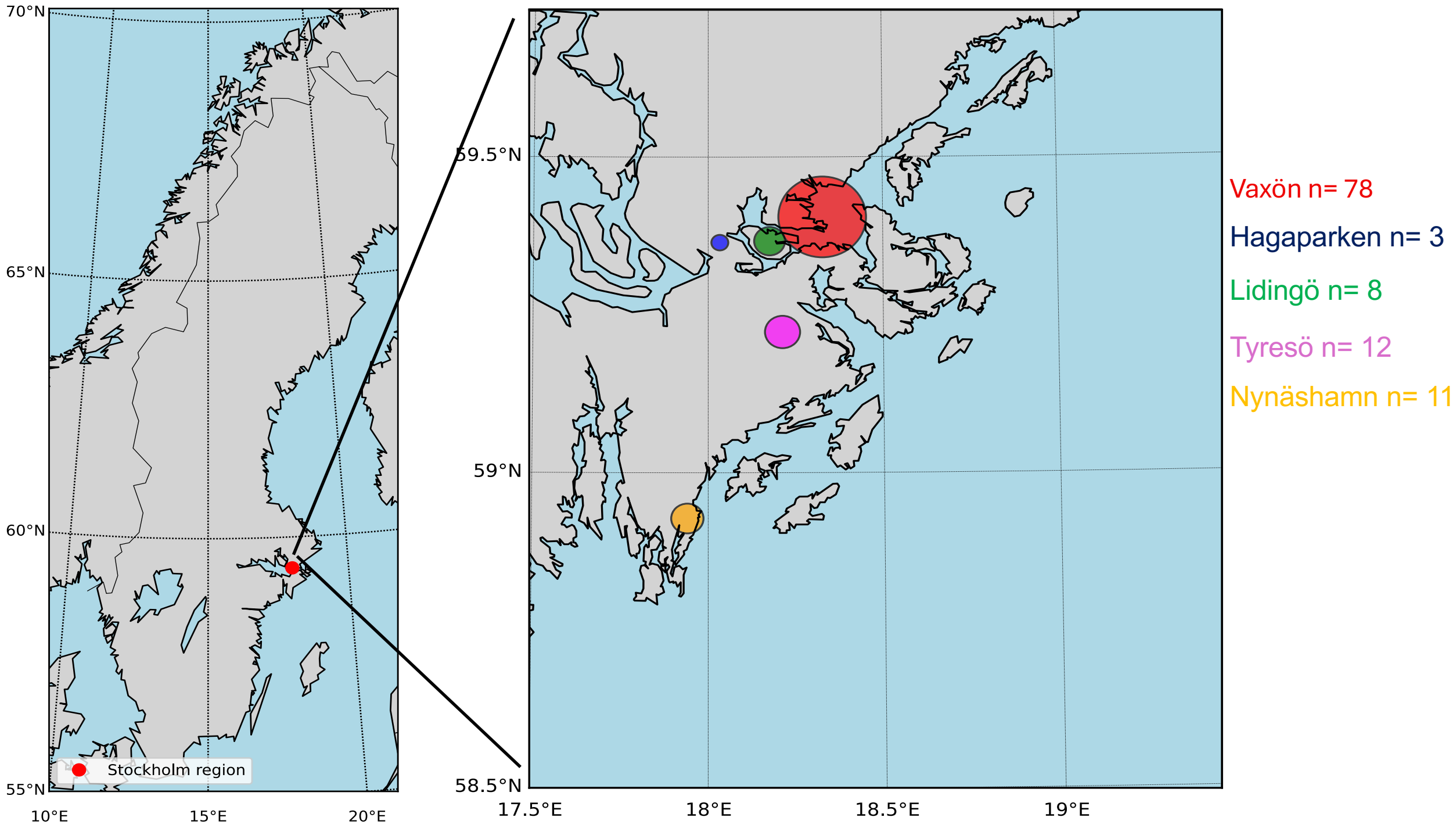
70°N
65°N
60°N
55°N
10°E
15°E
20°E
Stockholm region
59.5°N
59°N
58.5°N
17.5°E
18°E
18.5°E
19°E
Vaxön n= 78
Hagaparken n= 3
Lidingö n= 8
Tyresö n= 12
Nynäshamn n= 11

S1 Fig.
