# Supplementary material for: Life in sediments fosters ‘sexual’ speciation in the Shewanella baltica complex
Source: PLoS Genet. 2026 Sep 11;22(9):e1012306. doi: 10.1371/journal.pgen.1012306 (PMC13600613; doi:10.1371/journal.pgen.1012306)
Supplement: Supplementary file 2 [file pgen.1012306.s002.pdf]

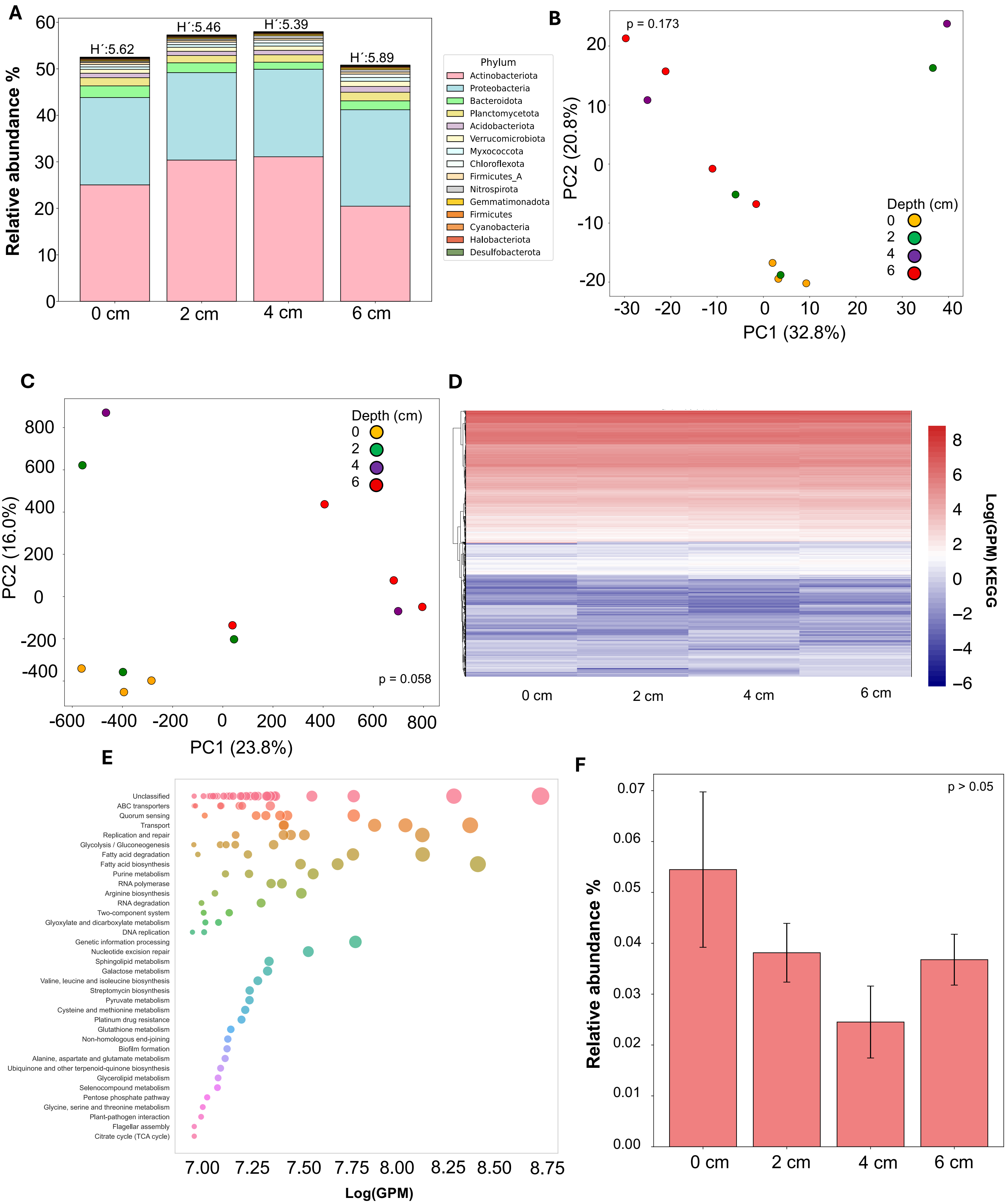

A
Relative abundance %
H´:5.62
H´:5.46
H´:5.39
H´:5.89
0 cm
2 cm
4 cm
6 cm
Phylum
Actinobacteriota
Proteobacteria
Bacteroidota
Planctomycetota
Acidobacteriota
Verrucomicrobiota
Myxococcota
Chloroflexota
Firmicutes_A
Nitrospirota
Gemmatimonadota
Firmicutes
Cyanobacteria
Halobacteriota
Desulfobacterota
B
p = 0.173
PC2 (20.8%)
PC1 (32.8%)
Depth (cm)
0
2
4
6
C
Depth (cm)
0
2
4
6
PC2 (16.0%)
PC1 (23.8%)
p = 0.058
D
0 cm
2 cm
4 cm
6 cm
Log(GPM) KEGG
E
Unclassified
ABC transporters
Quorum sensing
Transport
Replication and repair
Glycolysis / Gluconeogenesis
Fatty acid degradation
Fatty acid biosynthesis
Purine metabolism
RNA polymerase
Arginine biosynthesis
RNA degradation
Two-component system
Glyoxylate and dicarboxylate metabolism
DNA replication
Genetic information processing
Nucleotide excision repair
Sphingolipid metabolism
Galactose metabolism
Valine, leucine and isoleucine biosynthesis
Streptomycin biosynthesis
Pyruvate metabolism
Cysteine and methionine metabolism
Platinum drug resistance
Glutathione metabolism
Non-homologous end-joining
Biofilm formation
Alanine, aspartate and glutamate metabolism
Ubiquinone and other terpenoid-quinone biosynthesis
Glycerolipid metabolism
Selenocompound metabolism
Pentose phosphate pathway
Glycine, serine and threonine metabolism
Plant-pathogen interaction
Flagellar assembly
Citrate cycle (TCA cycle)
Log(GPM)
F
p > 0.05
Relative abundance %
0 cm
2 cm
4 cm
6 cm

S2 Fig.
