# Supplementary material for: Life in sediments fosters ‘sexual’ speciation in the Shewanella baltica complex
Source: PLoS Genet. 2026 Sep 11;22(9):e1012306. doi: 10.1371/journal.pgen.1012306 (PMC13600613; doi:10.1371/journal.pgen.1012306)
Supplement: Supplementary file 3 [file pgen.1012306.s003.pdf]

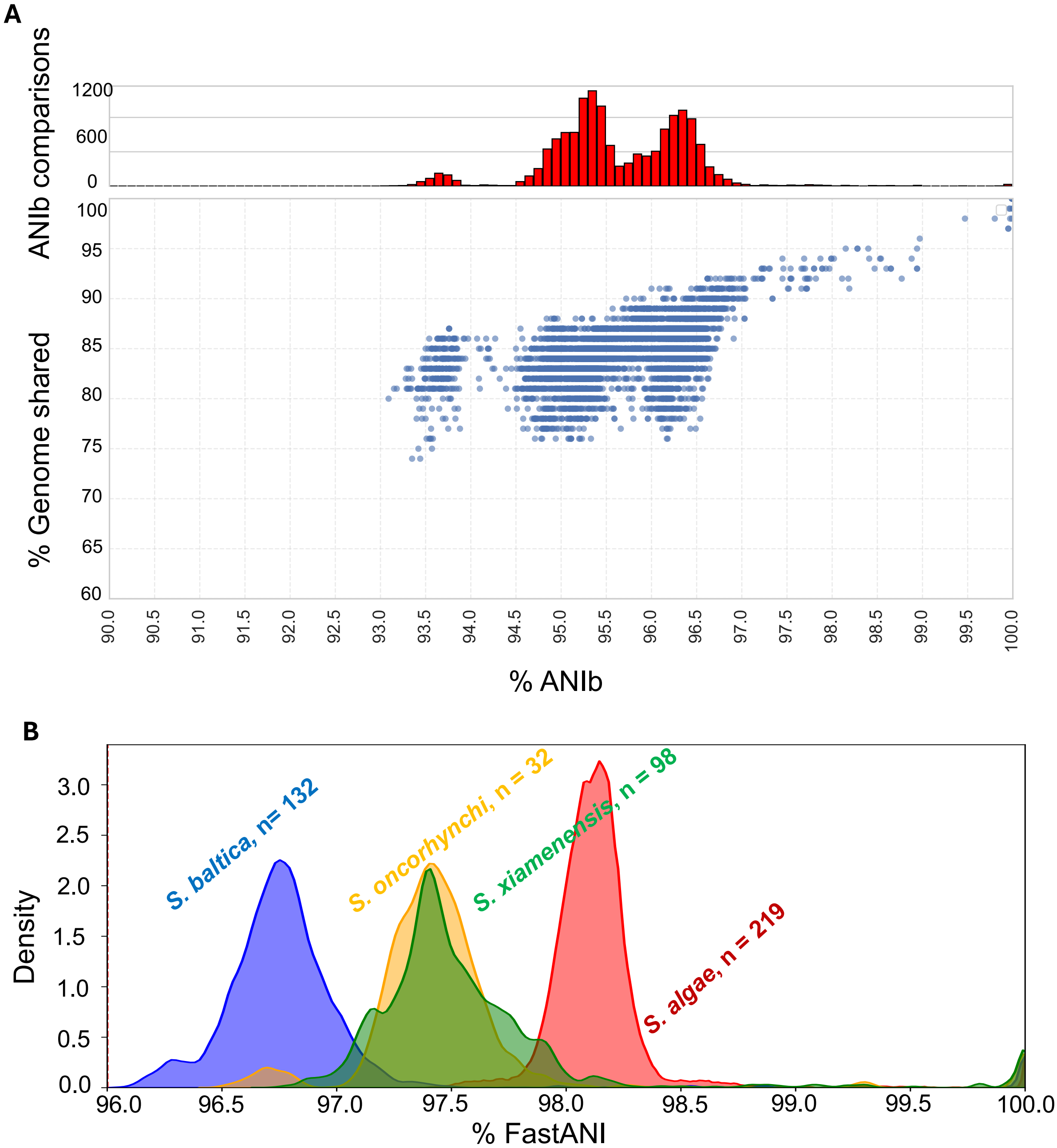
A
ANIb comparisons
1200
600
0
% Genome shared
100
95
90
85
80
75
70
65
60
90.0
90.5
91.0
91.5
92.0
92.5
93.0
93.5
94.0
94.5
95.0
95.5
96.0
96.5
97.0
97.5
98.0
98.5
99.0
99.5
100.0
% ANIb
B
Density
3.0
2.5
2.0
1.5
1.0
0.5
0.0
S. baltica, n= 132
S. oncorhynchi, n = 32
S. xiamenensis, n = 98
S. algae, n = 219
96.0
96.5
97.0
97.5
98.0
98.5
99.0
99.5
100.0
% FastANI

S3 Fig.
