# Supplementary material for: Life in sediments fosters ‘sexual’ speciation in the Shewanella baltica complex
Source: PLoS Genet. 2026 Sep 11;22(9):e1012306. doi: 10.1371/journal.pgen.1012306 (PMC13600613; doi:10.1371/journal.pgen.1012306)
Supplement: Supplementary file 4 [file pgen.1012306.s004.pdf]

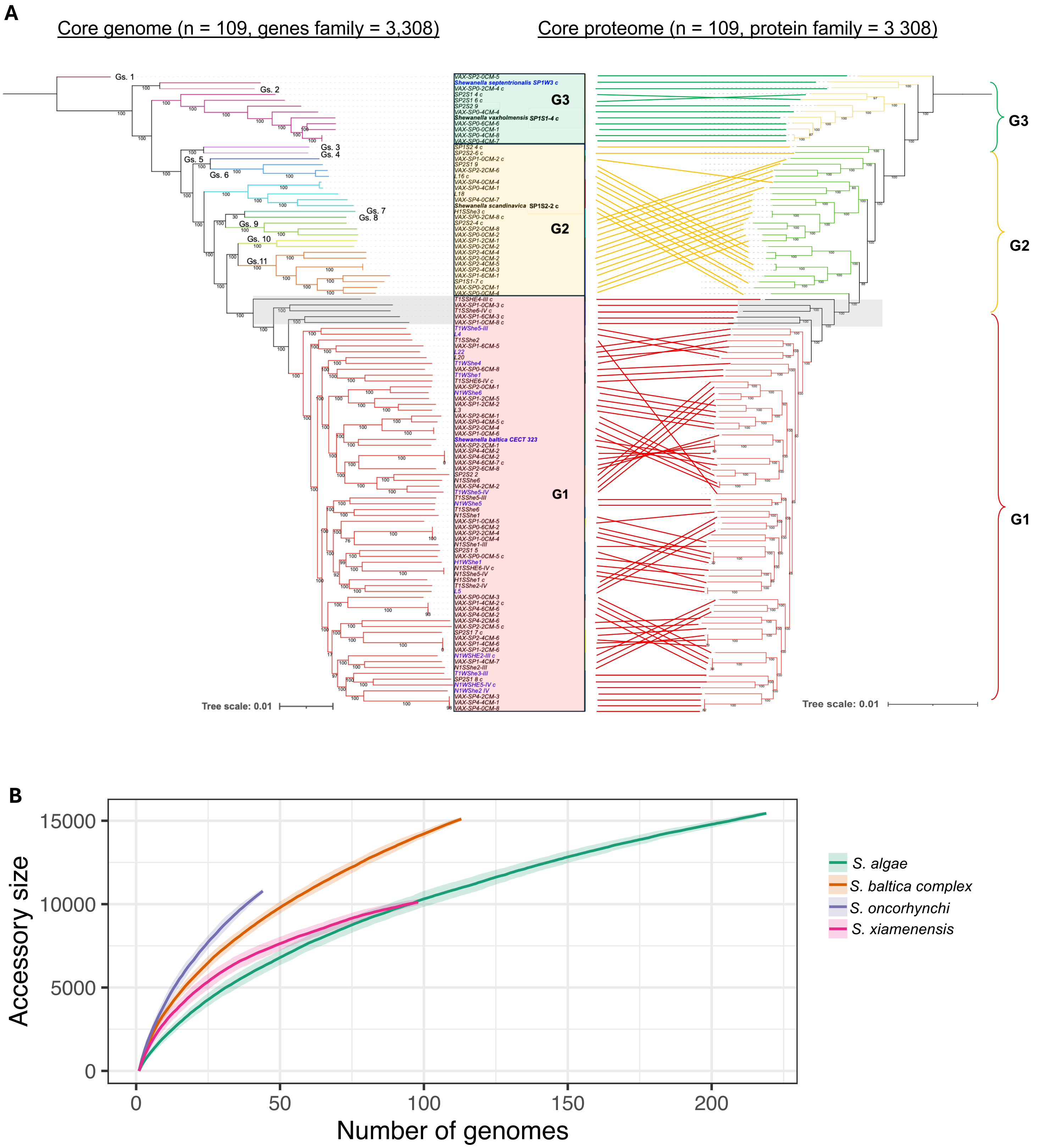
A
Core genome (n = 109, genes family = 3,308)
Core proteome (n = 109, protein family = 3 308)
G3
G2
G1
Tree scale: 0.01
Tree scale: 0.01
B
Accessory size
Number of genomes
S. algae
S. baltica complex
S. oncorhynchi
S. xiamenensis

S4 Fig.
