# Supplementary material for: Life in sediments fosters ‘sexual’ speciation in the Shewanella baltica complex
Source: PLoS Genet. 2026 Sep 11;22(9):e1012306. doi: 10.1371/journal.pgen.1012306 (PMC13600613; doi:10.1371/journal.pgen.1012306)
Supplement: Supplementary file 5 [file pgen.1012306.s005.pdf]

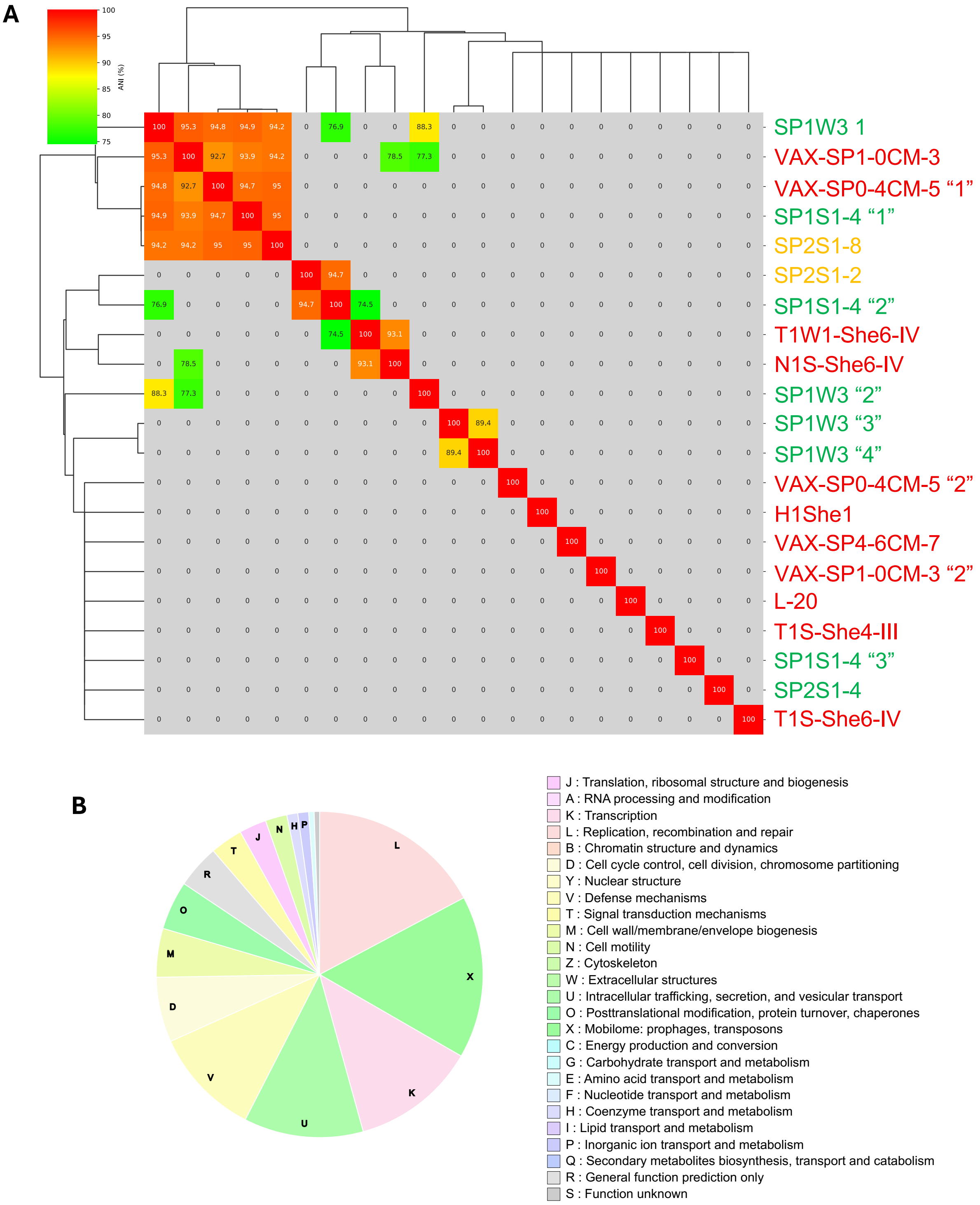
A
100
95
90
85
80
75
ANI (%)
SP1W3 1
VAX-SP1-0CM-3
VAX-SP0-4CM-5 “1”
SP1S1-4 “1”
SP2S1-8
SP2S1-2
SP1S1-4 “2”
T1W1-She6-IV
N1S-She6-IV
SP1W3 “2”
SP1W3 “3”
SP1W3 “4”
VAX-SP0-4CM-5 “2”
H1She1
VAX-SP4-6CM-7
VAX-SP1-0CM-3 “2”
L-20
T1S-She4-III
SP1S1-4 “3”
SP2S1-4
T1S-She6-IV
B
L
X
K
U
V
D
M
O
R
T
J
N
H
P
J : Translation, ribosomal structure and biogenesis
A : RNA processing and modification
K : Transcription
L : Replication, recombination and repair
B : Chromatin structure and dynamics
D : Cell cycle control, cell division, chromosome partitioning
Y : Nuclear structure
V : Defense mechanisms
T : Signal transduction mechanisms
M : Cell wall/membrane/envelope biogenesis
N : Cell motility
Z : Cytoskeleton
W : Extracellular structures
U : Intracellular trafficking, secretion, and vesicular transport
O : Posttranslational modification, protein turnover, chaperones
X : Mobilome: prophages, transposons
C : Energy production and conversion
G : Carbohydrate transport and metabolism
E : Amino acid transport and metabolism
F : Nucleotide transport and metabolism
H : Coenzyme transport and metabolism
I : Lipid transport and metabolism
P : Inorganic ion transport and metabolism
Q : Secondary metabolites biosynthesis, transport and catabolism
R : General function prediction only
S : Function unknown

S5 Fig.
