# Supplementary material for: Life in sediments fosters ‘sexual’ speciation in the Shewanella baltica complex
Source: PLoS Genet. 2026 Sep 11;22(9):e1012306. doi: 10.1371/journal.pgen.1012306 (PMC13600613; doi:10.1371/journal.pgen.1012306)
Supplement: Supplementary file 6 [file pgen.1012306.s006.pdf]

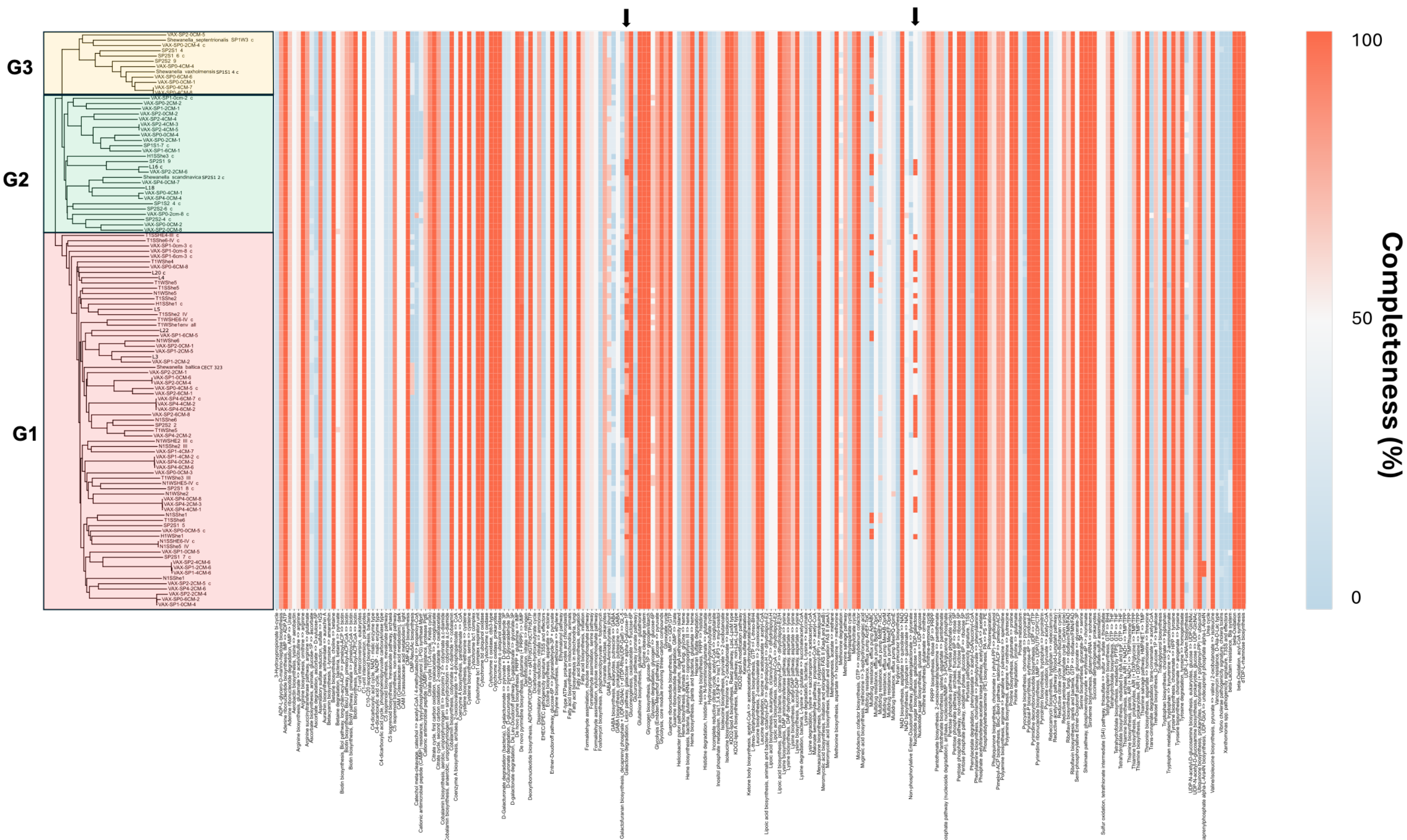

S6 Fig.
