# Supplementary material for: Life in sediments fosters ‘sexual’ speciation in the Shewanella baltica complex
Source: PLoS Genet. 2026 Sep 11;22(9):e1012306. doi: 10.1371/journal.pgen.1012306 (PMC13600613; doi:10.1371/journal.pgen.1012306)
Supplement: Supplementary file 7 [file pgen.1012306.s007.pdf]

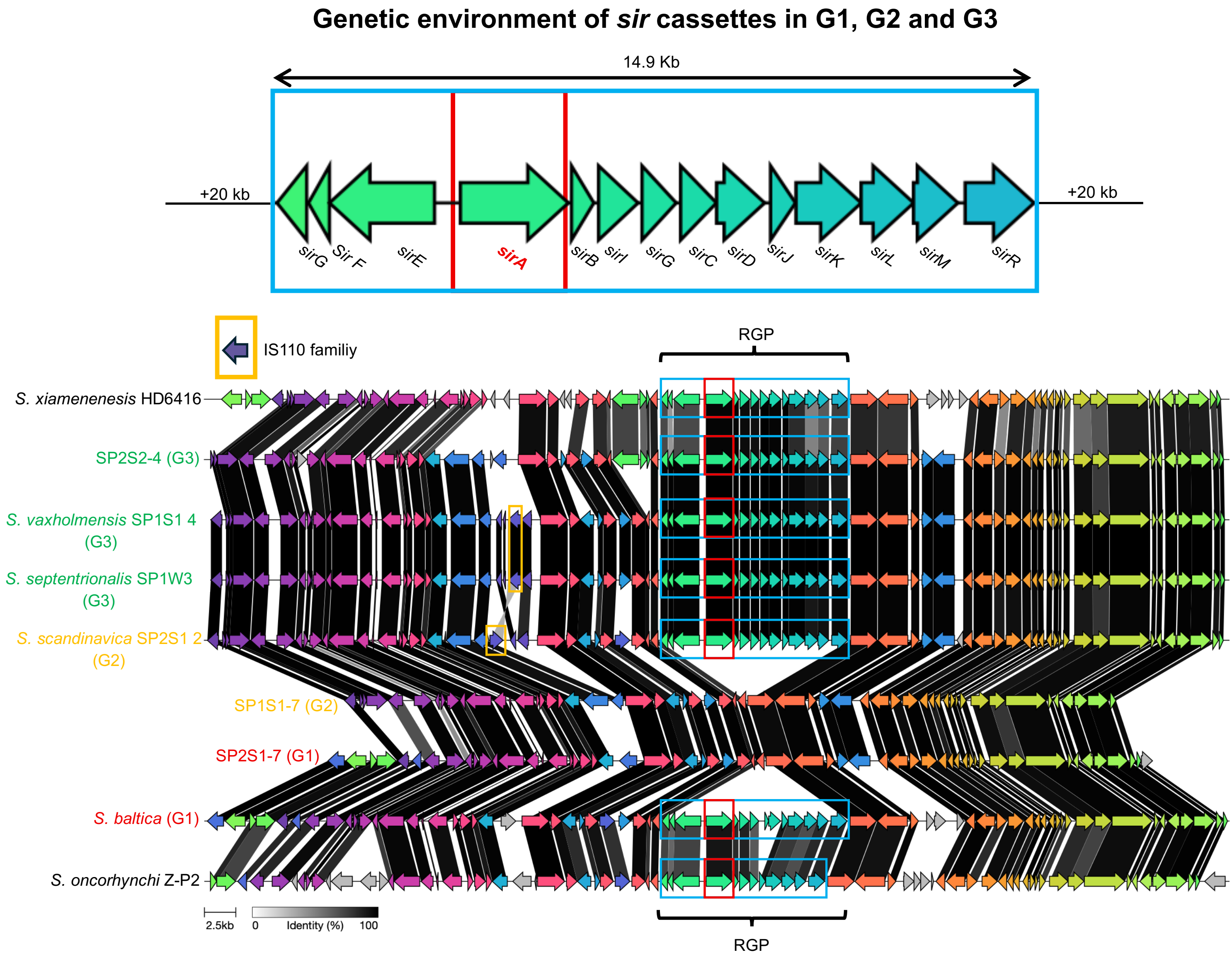

Genetic environment of *sir* cassettes in G1, G2 and G3
14.9 Kb
+20 kb
+20 kb
sirG
Sir F
sirE
sirA
sirB
sirI
sirG
sirC
sirD
sirJ
sirK
sirL
sirM
sirR
IS110 familiy
RGP
S. xiamenenesis HD6416
SP2S2-4 (G3)
S. vaxholmensis SP1S1 4 (G3)
S. septentrionalis SP1W3 (G3)
S. scandinavica SP2S1 2 (G2)
SP1S1-7 (G2)
SP2S1-7 (G1)
S. baltica (G1)
S. oncorhynchi Z-P2
2.5kb
0
Identity (%)
100
RGP

S7 Fig.
