# Supplementary material for: Life in sediments fosters ‘sexual’ speciation in the Shewanella baltica complex
Source: PLoS Genet. 2026 Sep 11;22(9):e1012306. doi: 10.1371/journal.pgen.1012306 (PMC13600613; doi:10.1371/journal.pgen.1012306)
Supplement: Supplementary file 8 [file pgen.1012306.s008.pdf]

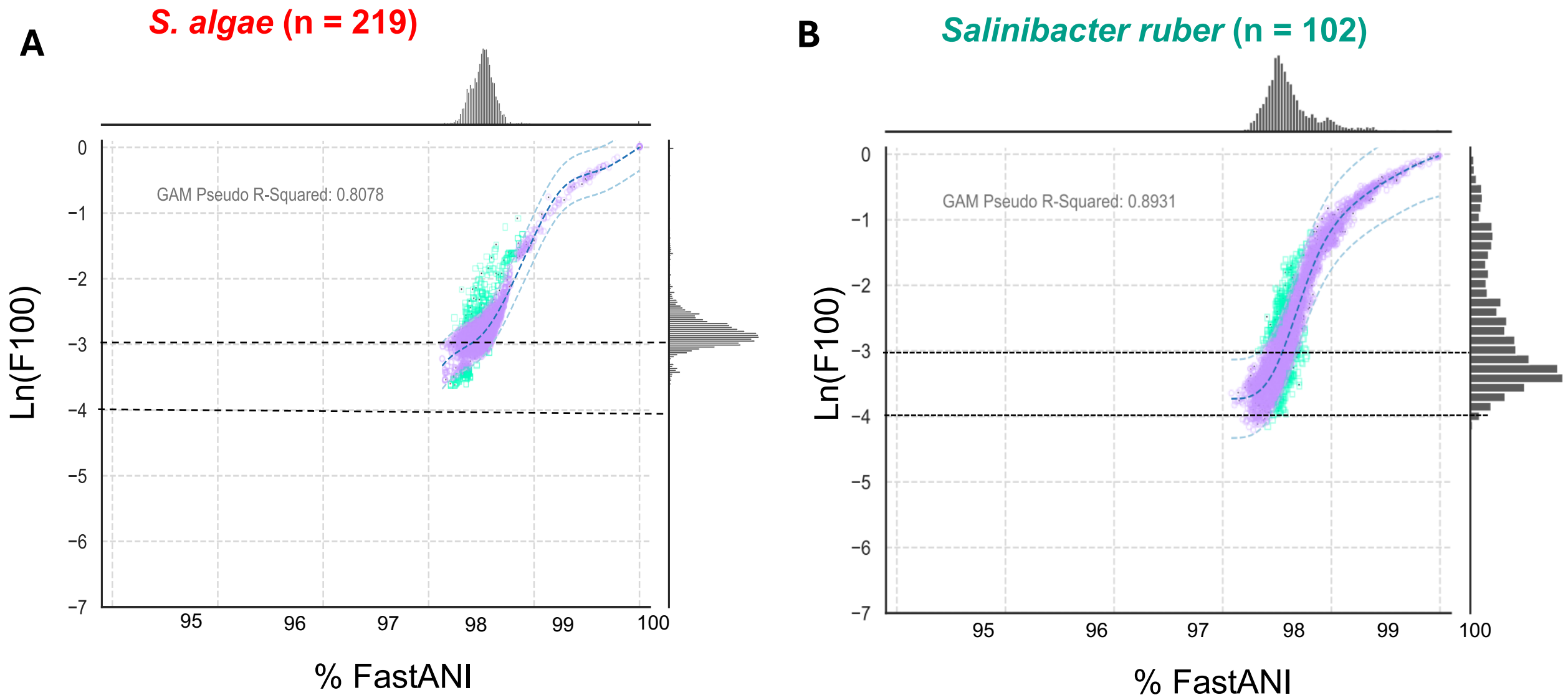
A
S. algae (n = 219)
GAM Pseudo R-Squared: 0.8078
Ln(F100)
% FastANI
B
Salinibacter ruber (n = 102)
GAM Pseudo R-Squared: 0.8931
Ln(F100)
% FastANI

S8 Fig.
