# Supplementary material for: Life in sediments fosters ‘sexual’ speciation in the Shewanella baltica complex
Source: PLoS Genet. 2026 Sep 11;22(9):e1012306. doi: 10.1371/journal.pgen.1012306 (PMC13600613; doi:10.1371/journal.pgen.1012306)
Supplement: Supplementary file 9 [file pgen.1012306.s009.pdf]

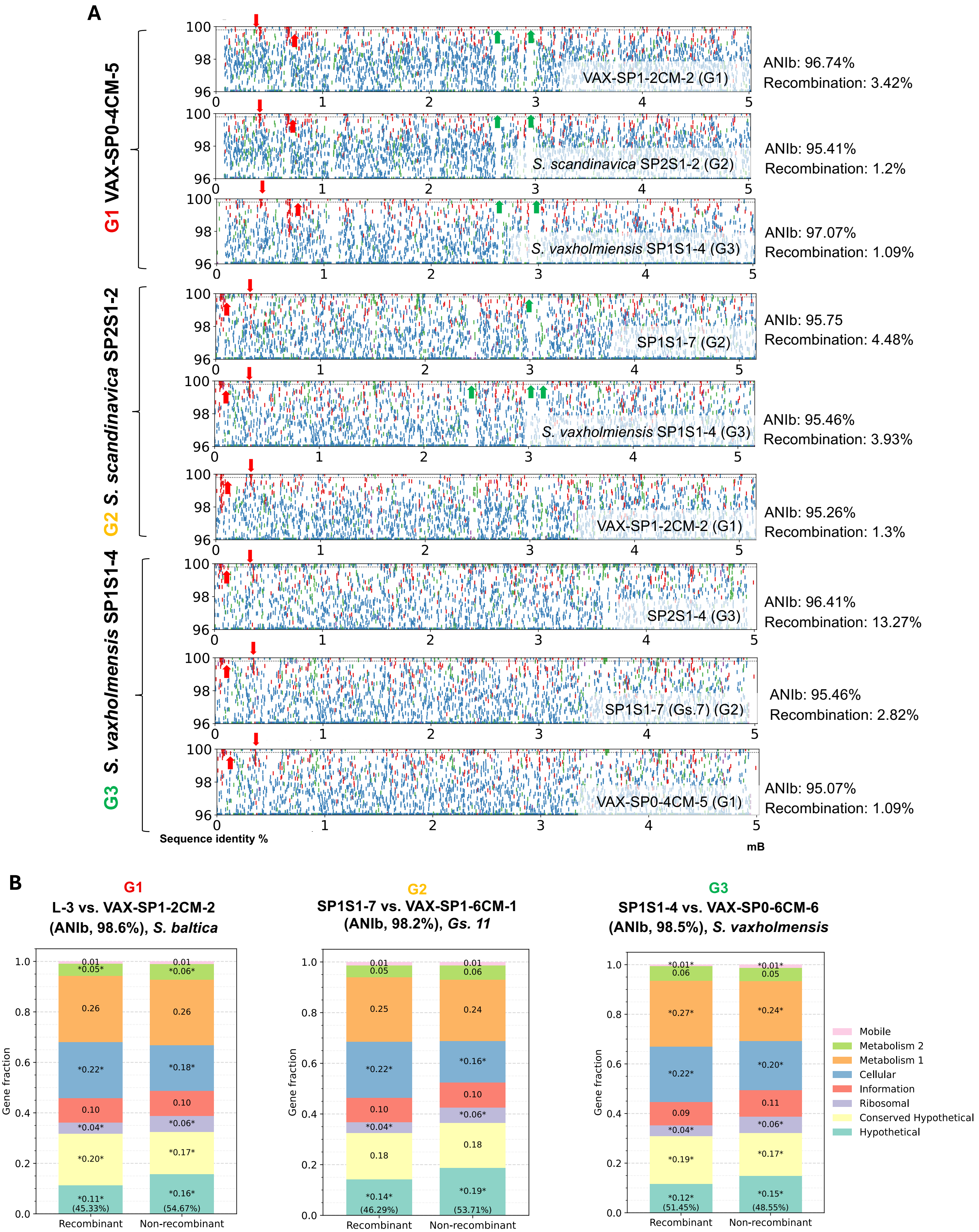

A
G1 VAX-SP0-4CM-5
VAX-SP1-2CM-2 (G1)
ANIb: 96.74%
Recombination: 3.42%
S. scandinavica SP2S1-2 (G2)
ANIb: 95.41%
Recombination: 1.2%
S. vaxholmiensis SP1S1-4 (G3)
ANIb: 97.07%
Recombination: 1.09%
G2 S. scandinavica SP2S1-2
SP1S1-7 (G2)
ANIb: 95.75
Recombination: 4.48%
S. vaxholmiensis SP1S1-4 (G3)
ANIb: 95.46%
Recombination: 3.93%
VAX-SP1-2CM-2 (G1)
ANIb: 95.26%
Recombination: 1.3%
G3 S. vaxholmensis SP1S1-4
SP2S1-4 (G3)
ANIb: 96.41%
Recombination: 13.27%
SP1S1-7 (Gs.7) (G2)
ANIb: 95.46%
Recombination: 2.82%
VAX-SP0-4CM-5 (G1)
ANIb: 95.07%
Recombination: 1.09%
Sequence identity %
mB
B
G1
L-3 vs. VAX-SP1-2CM-2
(ANIb, 98.6%), S. baltica
G2
SP1S1-7 vs. VAX-SP1-6CM-1
(ANIb, 98.2%), Gs. 11
G3
SP1S1-4 vs. VAX-SP0-6CM-6
(ANIb, 98.5%), S. vaxholmensis
Gene fraction
Recombinant
Non-recombinant
Mobile
Metabolism 2
Metabolism 1
Cellular
Information
Ribosomal
Conserved Hypothetical
Hypothetical

S9 Fig.
