# Supplementary material for: Life in sediments fosters ‘sexual’ speciation in the Shewanella baltica complex
Source: PLoS Genet. 2026 Sep 11;22(9):e1012306. doi: 10.1371/journal.pgen.1012306 (PMC13600613; doi:10.1371/journal.pgen.1012306)
Supplement: Supplementary file 10 [file pgen.1012306.s010.pdf]

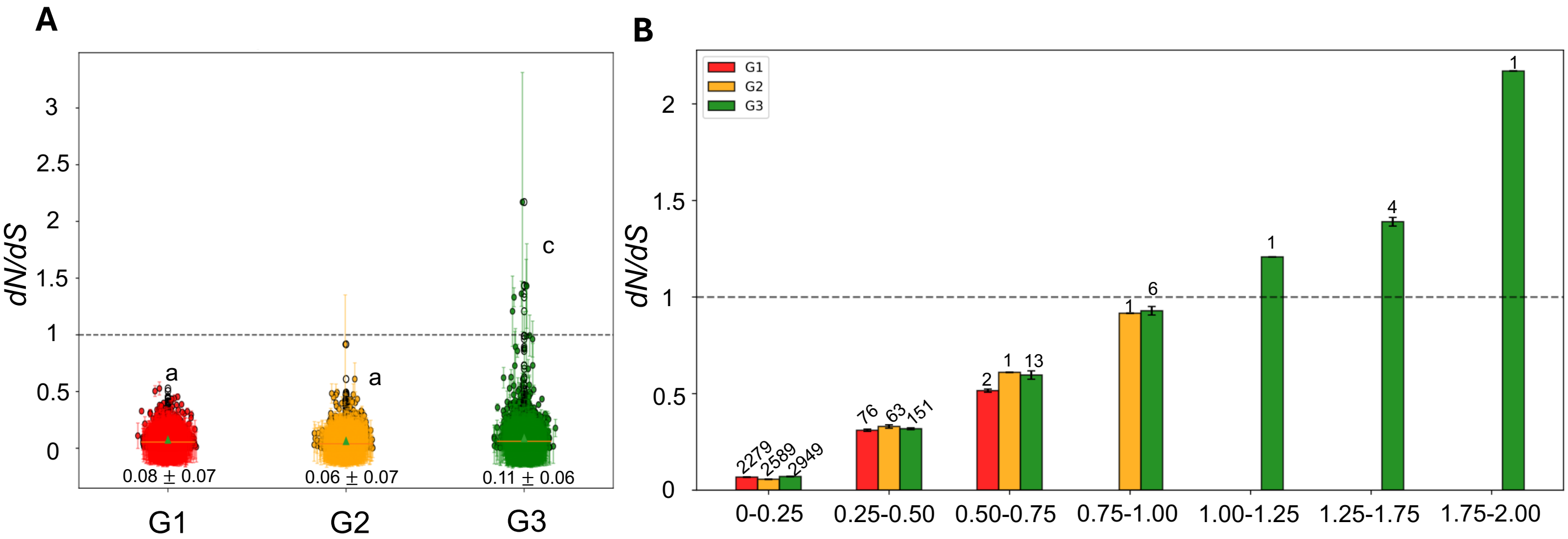
A
B
dN/dS
3
2.5
2
1.5
1
0.5
0
a
a
c
0.08 ± 0.07
0.06 ± 0.07
0.11 ± 0.06
G1
G2
G3
G1
G2
G3
2279
2589
2949
76
63
151
2
1
13
1
6
1
4
1
0-0.25
0.25-0.50
0.50-0.75
0.75-1.00
1.00-1.25
1.25-1.75
1.75-2.00

S10 Fig.
